# Combinatorial DNMTs and EZH2 inhibition reprograms the H3K27me3 and DNAme-mediated onco-epigenome to suppress multiple myeloma proliferation

**DOI:** 10.1038/s41598-025-17093-z

**Published:** 2025-08-27

**Authors:** Alba Atienza Párraga, Patrick Nylund, Klev Diamanti, Berta Garrido-Zabala, Stefania Iliana Tziola, Louella Vasquez, Paul Theodor Pyl, Doroteya Raykova, Aron Skaftason, Anqi Ma, Jian Jin, José Ignacio Martín-Subero, Fredrik Öberg, Elke De Bruyne, Jan Komorowski, Helena Jernberg Wiklund, Antonia Kalushkova

**Affiliations:** 1 Department of Immunology, Genetics and Pathology, Rudbeck Laboratory, Science for Life Laboratory, Uppsala University, Uppsala, Sweden; 2 Department of Cell and Molecular Biology, Science for Life Laboratory, Uppsala University, Uppsala, Sweden; 3 Department of Laboratory Medicine, Science for Life Laboratory, National Bioinformatics Infrastructure Sweden, Lund University, Lund, Sweden; 4 Department of Pharmaceutical Biosciences, Science for Life Laboratory, Uppsala University, Biomedical center, Uppsala, Sweden; 5 Mount Sinai Center for Therapeutics Discovery, Departments of Pharmacological Sciences, Oncological Sciences and Neuroscience, Tisch Cancer Institute, Icahn School of Medicine at Mount Sinai, New York, NY 10029 USA; 6 Department of Pathology, Hematopathology Section, Institut d’Investigacions Biomèdiques August Pi i Sunyer (IDIBAPS), Hospital Clinic, University of Barcelona, Barcelona, Spain; 7 Institució Catalana de Recerca i Estudis Avançats (ICREA), Barcelona, Spain; 8 Medivir AB, Huddinge, Sweden; 9 Translational Oncology Research Center (TORC) – Team Hematology and Immunology (HEIM), Vrije Universiteit Brussel, Brussels, Belgium; 10 Institute of Computer Science, Polish Academy of Sciences, Warsaw, Poland; 11 Washington National Primate Research Center, Seattle, WA USA; 12 Swedish Collegium for Advanced Study, Uppsala, Sweden

**Keywords:** Cancer, Cell biology

## Abstract

Comprehensive epigenomic studies in multiple myeloma (MM) that unravel the connections between major epigenetic regulators, their intertwined collaboration and the potential of combinatorial targeting remain limited. Utilizing ChIP-seq, ATAC-seq, RNA-seq, and DNA methylation (DNAme) data, we generated whole-genome chromatin annotations from normal plasma cells and MM patients, revealing epigenomic re-configuration affecting downstream genes involved in tumour growth and survival. Primary MM samples showed global DNA hypomethylation but site-specific hypermethylation was observed at transcription start sites, promoters, and enhancers. Moreover, increased deposition of H3K27me3 was observed in clinically relevant functional chromatin clusters. Combined EZH2 and DNMTs inhibition resulted in extensive epigenomic alterations activating apoptosis and cell cycle genes, leading to increased G2/M arrest and apoptosis in MM cell lines. Our findings provide novel insights into the role of epigenetic gene silencing in MM tumorigenesis and the interplay between the Polycomb repressive complex 2 and DNAme.

**Supplementary Information:**

The online version contains supplementary material available at 10.1038/s41598-025-17093-z.

## Introduction

Multiple myeloma (MM) is characterised by the accumulation of malignant cells with plasmablast/plasma cell-like features in the bone marrow^1^. Extensive genetic heterogeneity has hampered the identification of common mechanisms amenable to therapeutic intervention^2^. Therefore, MM patients often develop drug resistance to conventional treatments and eventually relapse^3^. Emerging evidence has highlighted the vital role of epigenetic aberrations in MM, including DNA methylation (DNAme) and histone post-translational modifications^4–11^.

We previously identified a Polycomb repressive complex 2 (PRC2)-mediated gene repression profile as a common hallmark of MM^12^. Genome-wide mapping of genes marked by histone H3 lysine 27 trimethylation (H3K27me3) revealed that their silencing strongly correlated with advanced stages and poor clinical outcome^13^. Furthermore, active enhancers in normal plasma cells (NPCs) become DNA methylated, and lose their enhancer-defining H3K27ac and H3K4me1 marks in MM, a process which is associated with loss of activity of transcription factors (TFs) that guide B cell differentiation^5^. These loci were found to be highly methylated in embryonic stem cells and immature B cells and became gradually demethylated during B cell maturation. Similar to findings linking PRC2-mediated silencing to the preservation of a less differentiated state in tumours^14^, B cell enhancer hypermethylation may further contribute to maintaining stemness features in MM^15^. The transition from the premalignant stage of monoclonal gammopathy of undetermined significance (MGUS) to MM is characterised by genome-wide DNA hypomethylation and gene-specific hypermethylation, whereas extensive DNA re-methylation is prominent in the transition from MM to plasma cell leukaemia^4^. Furthermore, among the genes transcriptionally repressed through DNAme in MM, many have a tumour suppressor function^16^. As such, inhibition of the DNMTs is a promising candidate for therapeutic intervention. Treatment with the DNMTs inhibitor 5-azacytidine has been shown to sensitize MM cells to conventional therapies such as bortezomib^17^ and is currently FDA-approved for treatment of several other haematological disorders^18^.

Epigenetic regulation of gene expression often stems from the cooperation of several components. Although the precise relationship between them remains unclear, the catalytic component of PRC2, namely EZH2, has been shown to directly interact with DNA methyltransferases in leukemic cells^19^ and to mark a set of genes that gain aberrant DNAme during malignant transformation in colon cancer^20^. In MM, although a direct interaction has not yet been proven, both EZH2 and DNMT1 are overexpressed as compared to NPCs^12,16^. Furthermore, other studies suggest that a subset of PRC2-regulated promoters are highly DNA-methylated in MM^21^.

In this paper, we generated new epigenomic data highlighting that, compared to NPCs, malignant plasma cells of MM undergo a widespread reconfiguration of H3K27me3 and DNAme. Moreover, we demonstrated a physical interaction between DNMT1 and EZH2 in MM cells, indicating an interplay between these two epigenetic silencers. To unravel the downstream effects of their dual inhibition, we generated ChIP-seq, RNA-seq and DNAme arrays data on samples treated with a combination of the EZH2 inhibitor UNC1999^22^ and the hypomethylating agent 5-azacytidine^23^. As a result, we show that the combinatorial treatment leads to reduced cell viability through activation of genes associated with apoptosis and cell cycling in MM.

## Results

### Treatment with 5-azacytidine promotes the activation of pro-apoptotic genes through loss of DNA methylation at promoter regions

**Fig. 1:**
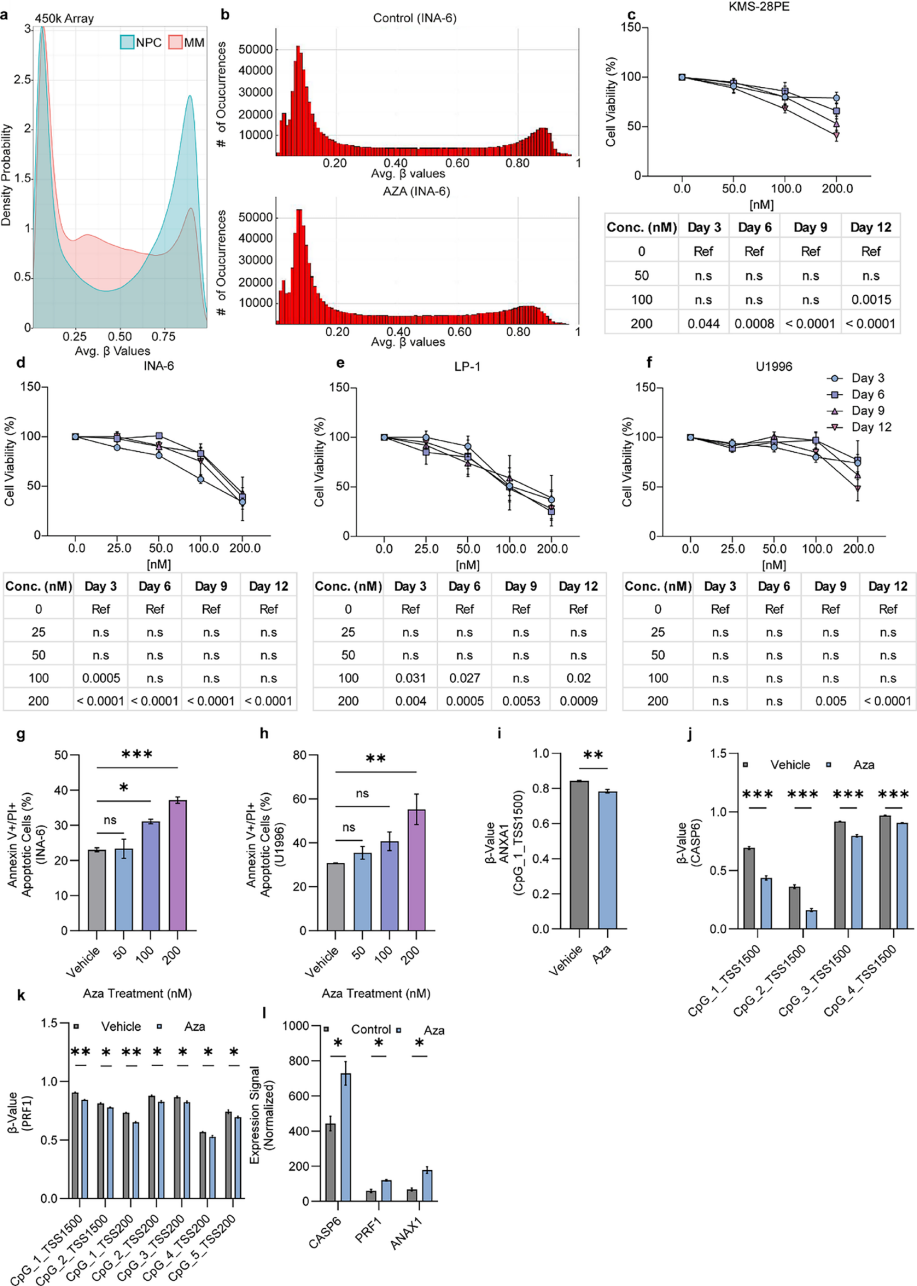
Prolonged low-dose 5-azacytidine treatment activates apoptosis-associated genes but has limited effects on MM viability in vitro. **(a)** DNA methylation differences between normal plasma cells (NPCs) (*n* = 8) and MM patients (*n* = 104). **(b)** DNA methylation profile in the MM cell line INA-6 treated with 50nM 5-azacytidine (AZA) for 9 days. **(c-f)** Viability analysis upon 12 days of treatment with AZA in the MM cell lines (c) KMS28-PE, (d) INA-6, (e) LP-1 and (f) U1996. Tables demonstrate statistical analysis (i.e. p-value) of difference in cell viability between treatment and vehicle at each timepoint per cell line. Statistical analysis was performed with two-way ANOVA and corrected for multiple testing by Tukey-test. Values presented with SEM. **(g-h)** Apoptosis analysis upon 6 days of AZA treatment in the (g) INA-6 and (h) U1996 MM cell lines. Statistical analysis was performed with one-way ANOVA and corrected for multiple testing by Dunnett-test. Values presented with SEM. Corresponding gating can be found in supplementary Figs. 9–10. **(i-k)** Treatment of the INA-6 MM cells with 50nM of AZA leads to loss of DNA methylation around TSS areas of the apoptosis-associated genes (i) *ANXA1*, (j) *CASP6* and (k) *PRF1*. Statistical analysis was performed with (i) unpaired t-test, (j-k) multiple unpaired t-test, corrected for multiple testing with Holm-Šídák method. Values presented with SEM. **(l)** Treatment with 50nM of AZA leads to gain of expression of *ANXA1*, *CASP6* and *PRF1* in the INA-6 MM cells. Statistical analysis was performed with multiple unpaired t-test, corrected for multiple testing with Holm-Šídák method. Values presented with SEM. All experiments were conducted in three biological replicates, *p < 0.05, **p < 0.01, ***p < 0.001, ****p < 0.0001.

Although MM cells undergo a global hypomethylation as compared to NPCs, they do harbour site-specific hypermethylation (Fig. 1a) affecting regulatory elements^5^. To explore the functional impact of local hypermethylation, we investigated the effects of reducing DNAme using the FDA-approved cytosine analogue 5-azacytidine^23^. The effects of the drug have been investigated in a variety of contexts, however, many of these report cytotoxic effects due to its intercalation into the DNA when used at high doses^24^. Therefore, we aimed to employ a prolonged low-dose treatment strategy that more closely resembles clinical exposure conditions^25^, with the goal of inducing DNA demethylation and investigating its effects on MM cells. Treatment with doses as low as 12.5 nM resulted in an efficient reduction of DNMT1 and DNMT3A protein levels after six days of treatment, which persisted throughout the duration of the experiment (i.e., 12 days) in all investigated MM cell lines (Supplementary Fig. 1a). In addition, analysis of global DNAme patterns using high-density microarray (Illumina Infinium MethylationEPIC) in the 5-azacytidine-treated MM cell line INA-6 confirmed that the reduction in DNMT proteins levels was accompanied by decreased DNAme in regions previously exhibiting a hypermethylation profile (Fig. 1b). To evaluate the subsequent effects on MM cells, 5-azacytidine-treated samples were subjected to viability and apoptosis evaluation. The investigated MM cell lines displayed heterogeneous sensitivity to the demethylating treatment (Fig. 1c-f). Sensitivity at higher concentrations (100–200 nM) was accompanied by the induction of apoptosis (Fig. 1g-h). Lower doses (50 nM) of 5-azacytidine were insufficient to induce a significant increase in apoptotic cells but were able to re-activate the expression of the pro-apoptotic genes *PRF1*, *CASP6* and *ANXA1* through DNAme loss (Fig. 1i-l).

These results demonstrate that while 5-azacytidine treatment reduced tumour cell viability at higher doses, a prolonged low-dose approach can effectively reduce DNMT levels and cause DNA demethylation without causing major alterations in cell viability, thus setting the stage for exploration of a combinatorial treatment strategy.

### Extensive reconfiguration of the chromatin landscape between normal and malignant plasma cells reveals novel therapeutic targets

**Fig. 2:**
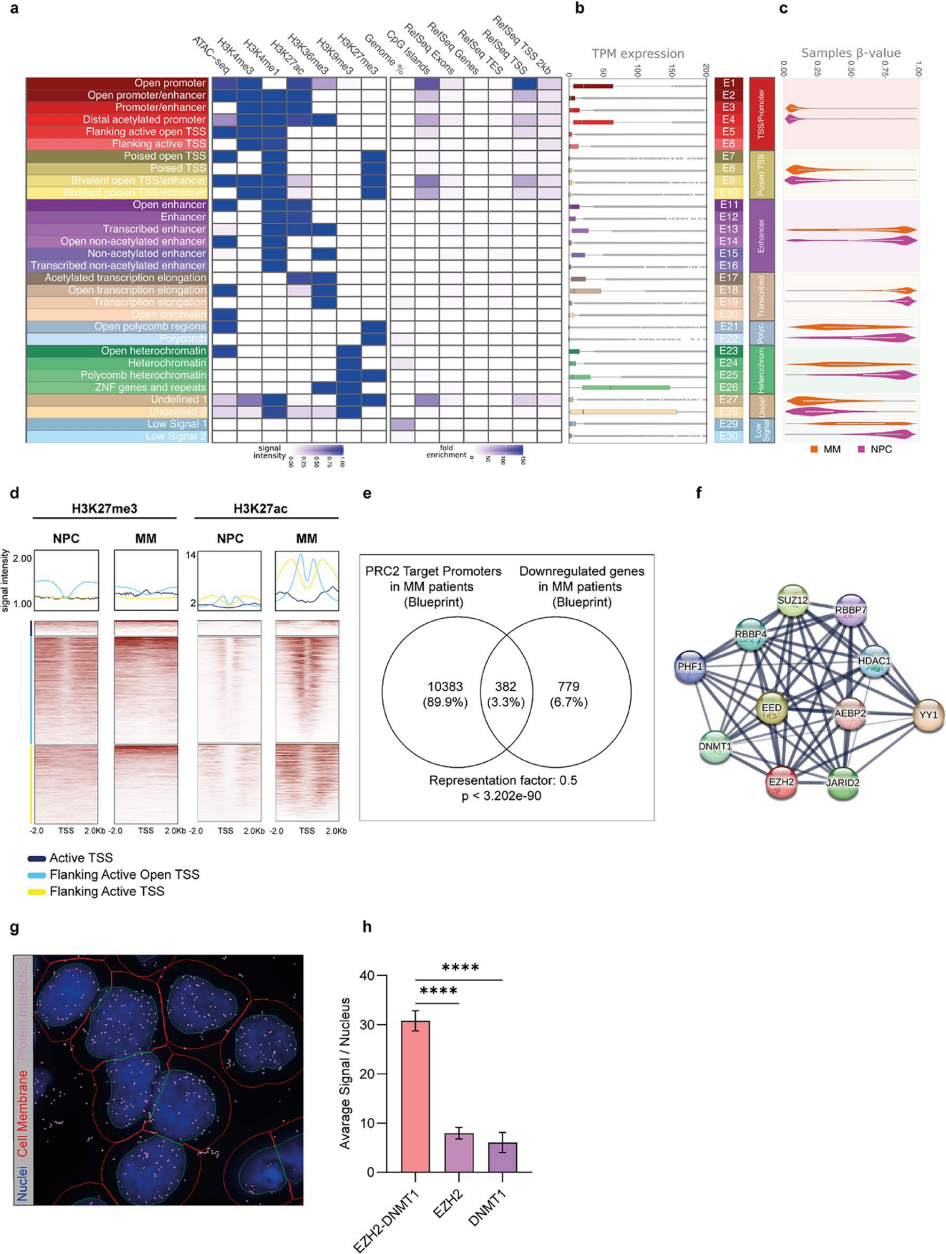
Epigenomic reconfiguration between normal plasma cells (NPCs) and MM patient cells leads to the identification of EZH2 and DNMT1 as interacting partners in MM. **(a)** Thirty clusters of regulatory elements identified by ChromHMM based on the combination of H3K4me1, H3K4me3, H3K9me3, H3K27ac, H3K27me3, H3K36me3 ChIP-seq and ATAC-seq signals. Summary of primary samples from the Blueprint Consortium can be found in supplementary Table I. **(b)** RNA-seq data for each cluster. ChromHMM clusters were further merged into eight cluster families. **(c)** Average DNA methylation values in NPCs and MM patients over the NPC-defined genomic regions (n_tPC_=8, n_MM_=104). **(d)** Top: average signal of ChIP-seq reads aligned around the centre of genomic regions belonging to the active TSS clusters. The colour of the lines represents the regulatory cluster. Bottom: signal-intensity-ordered heatmaps of each mark in NPCs and MM, aligned around the centre of genomic regions. Summary of primary samples from the Blueprint Consortium can be found in supplementary Table I. **(e)** Overlap between PRC2 target genes in MM patients marked with H3K27me3 and downregulated genes in MM vs. NPCs. **(f)** Protein-protein interaction network of EZH2 (https://string-db.org/). **(g)** Representative image showing protein-protein interaction between EZH2 and DNMT1 in the INA-6 MM cell line. Blue: nucleus, red: cell membrane and pink: EZH2-DNMT1 protein-protein interaction. **(h)** Average number of EZH2-DNMT1 interactions per cell in the INA-6 MM cell line compared to background (single-protein control staining). Statistical analysis was performed with one-way ANOVA and corrected for multiple testing by Dunnett-test. Values presented with SEM. Proximity ligation assay was conducted in three biological replicates, ****p < 0.0001.

To investigate the interplay between different epigenetic mechanisms in MM, we applied a hidden Markov model approach (ChromHMM)^26^ to segment the genome of NPCs and annotate functional regions based on combinations of histone marks and chromatin accessibility (Fig. 2a). Specifically, we used ChIP-seq data for H3K4me1, H3K4me3, H3K9me3, H3K27ac, H3K27me3, H3K36me3, along with ATAC-seq data from tonsillar plasma cells (tPCs) from five donors, available through the Blueprint Consortium (Supplementary Table I)^8,27^. A total of 30 distinct clusters of chromatin states were identified based on various combinations of these marks and subsequently grouped into 8 functional categories (i.e., TSS/promoter, poised TSS, enhancer, transcribed, Polycomb, heterochromatin, undefined and low signal) (Fig. 2a)^28–30^. In agreement with our classification, TSS/promoter regions were enriched for RefSeq TSS and RefSeq TSS ± 2 kb annotations (Fig. 2a). Moreover, we observed a significant overlap between ChromHMM-defined TSS regions and FANTOM5 TSSs (Supplementary Fig. 1b), as well as between enhancer regions and FANTOM5-defined enhancers^31^ (Supplementary Fig. 1c). Gene expression analysis from RNA-seq data in the same donor cohort further confirmed that poised TSS and Polycomb regions corresponded to some of the lowest-expressing loci (Fig. 2b).

The newly generated map of chromatin states and gene expression profiles in NPCs enabled us to investigate genome-wide differences in histone modification patterns between NPCs and MM by integrating ChIP-seq, ATAC-seq, and DNAme array data from primary MM samples (Supplementary Table I). In TSS/promoters, DNAme levels remained consistently low in both NPCs and MM, whereas most enhancers and transcribed regions displayed high levels of DNAme (Fig. 2c). MM patient samples exhibited extensive chromatin reprogramming across several chromatin states defined in NPCs (Supplementary Fig. 2–6), including regions annotated as Polycomb regions (Supplementary Fig. 2). Interestingly, heterochromatic regions initially marked by H3K9me3 in NPCs showed a switch to H3K27me3 in MM, whereas a proportion of Polycomb regions presented with the reciprocal switch (Supplementary Figs. 3). Within some enhancer regions, the increase in H3K27me3 was also accompanied by increased deposition of H3K27ac, reduction of H3K4me1 and elevated RNA-seq signal, suggesting that while some loci became transcriptionally silenced, others became highly active in MM (Supplementary Fig. 4–5). Furthermore, we identified a genome-wide H3K27ac enrichment in combination with local H3K27me3 and H3K9me3 enrichment at active TSS and promoter regions (Supplementary Fig. 6), indicating reprogramming of activating and repressive marks in MM (Fig. 2d). Interestingly, gene-set enrichment analysis (GSEA) of genes that were downregulated and marked by H3K27me3 in MM patients revealed enrichment for IL-2-STAT5 signalling (Fig. 2e) (Supplementary Fig. 7a).

Additionally, Polycomb and heterochromatin regions showed reduced DNAme in MM (Supplementary Fig. 7b). In contrast, loci classified as promoter/TSS regions, poised TSS, and enhancers in NPCs exhibited higher median DNAme levels in MM compared to NPCs (Supplementary Fig. 7b).

Intrigued by the extensive chromatin changes induced by PRC2 and DNAme, we next aimed to evaluate the relationship between these two epigenetic mechanisms in MM. Protein interaction data from STRING database (https://string-db.org/) suggested that DNMTs may functionally collaborate with EZH2 (Fig. 2f). In line with this, in situ proximity ligation assay (PLA) (Fig. 2g-h) and co-immunoprecipitation (Co-IP) (Supplementary Fig. 7c) confirmed a protein-protein interaction between these two enzymes in MM cell lines.

Taken together, these data illustrate that the chromatin landscape is extensively reprogrammed in MM, with certain genomic elements such as active TSSs and active enhancers acquiring repressive marks, while others becoming *de novo* activated. The redistribution of histone marks and changes in the DNA methylome, along with the physical interaction between the catalytic subunit of PRC2, EZH2 and DNMT1 supports the rationale for a therapeutic strategy of combining EZH2 and DNMTs inhibition as a potential clinical approach in MM.

### Dual Inhibition of EZH2 and DNMTs promotes global redistribution of chromatin marks and activation of tumour suppressor genes in MM

**Fig. 3:**
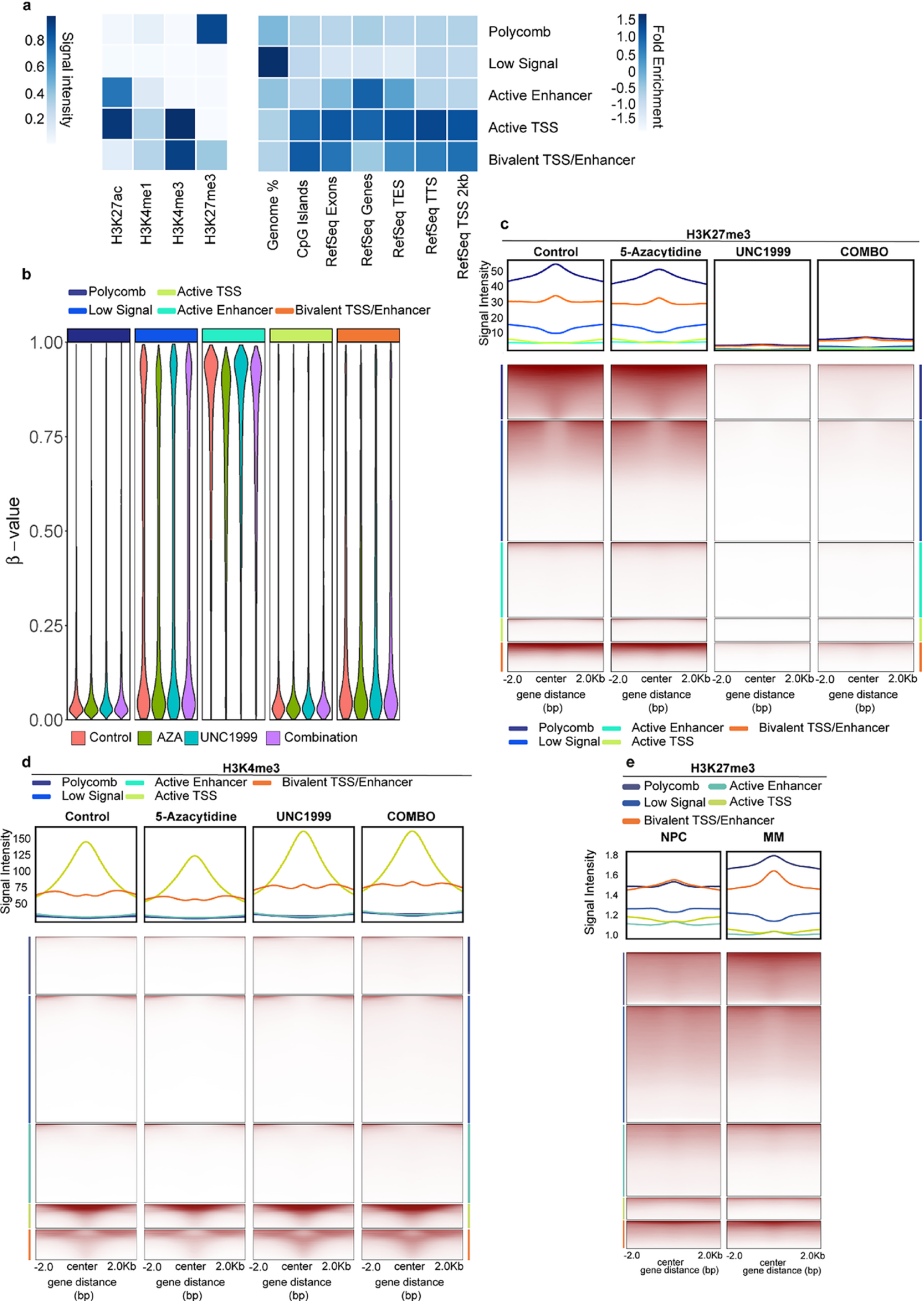
UNC1999 alone or in combination with 5-azacytidine (AZA) effectively reduces H3K27me3 in all ChromHMM-defined clusters, whereas regions characterised as active TSS gain H3K4me3. **(a)** Five clusters of regulatory elements identified by ChromHMM based on the combination of H3K4me1, H3K4me3, H3K27ac and H3K27me3 ChIP-seq signals in the vehicle-treated INA-6 MM cell line. All ChIP-seq experiments were conducted in minimum three biological replicates. **(b)** DNA methylation alterations in ChromHMM-defined clusters in the INA-6 MM cells treated with either vehicle, UNC1999, AZA or the combination of UNC1999 and AZA for a total of 9 days. DNA methylation array (Illumina Infinium MethylationEPIC) was conducted in three biological replicates. **(c)** Top: average signal of H3K27me3 ChIP-seq reads in control and treated INA-6 MM cells, aligned around the centre of genomic regions belonging to the Polycomb, active enhancer, active TSS and bivalent TSS/enhancer clusters. The colour of the lines represents regulatory clusters as indicated. Bottom: signal-intensity-ordered heatmaps of the H3K27me3 reads in control and treated INA-6 MM cells, aligned around the centre of the genomic regions. The INA-6 MM cells were treated with either vehicle, UNC199, AZA or the combination of UNC1999 and AZA for a total of 9 days. All ChIP-seq experiments were conducted in minimum three biological replicates.  **(d)** Top: average signal of H3K4me3 ChIP-seq reads in control and treated INA-6 MM cells, aligned around the centre of genomic regions belonging to the Polycomb, active enhancer, active TSS and bivalent TSS/enhancer clusters. The colour of the lines represents regulatory clusters as indicated. Bottom: signal-intensity-ordered heatmaps of H3K4me3 reads in control and treated INA-6 MM cells, aligned around the centre of the genomic regions. The INA-6 MM cells were treated with either vehicle, UNC199, AZA or the combination of UNC1999 and AZA for a total of 9 days. All ChIP-seq experiments were conducted in minimum three biological replicates. **(e)** Top: average signal of H3K27me3 ChIP-seq reads aligned around the centre of genomic regions belonging to the Polycomb, active enhancer, active TSS and bivalent TSS/enhancer clusters in normal plasma cells (NPCs) and MM patients. The colour of the lines represents regulatory clusters as indicated. Bottom: signal-intensity-ordered heatmaps of H3K27me3 reads in NPCs and MM patients, aligned around the centre of the genomic regions. Summary of primary samples from the Blueprint Consortium can be found in supplementary Table I.

We have previously demonstrated the impact of EZH2 inhibition on MM^32,33^. Given the observed physical interaction between EZH2 and DNMT1, we next sought to investigate the effects of their combined inhibition on the MM epigenome. We evaluated the genome-wide distribution of H3K27me3, H3K27ac, H3K4me1 and H3K4me3 by ChIP-seq, and high-density DNAme array (Illumina Infinium MethylationEPIC) in MM cells treated with the EZH2 inhibitor UNC1999, the DNAme inhibitor 5-azacytidine, or their combination. To evaluate changes in chromatin mark enrichment, we applied ChromHMM^26^ and identified five regulatory clusters we defined as Polycomb, active TSS, active enhancer, bivalent TSS/enhancer and low signal, based on combinations of histone marks in the control-treated samples (Fig. 3a). Regions defined as active TSS were further enriched for RefSeq TSS, RefSeq TSS 2 kb and CpG islands (Fig. 3a). After defining these functional genomic clusters, we examined DNAme distribution following treatment and observed global loss of DNAme in cells treated with 5-azacytidine alone or in combination with UNC1999 (Supplementary Fig. 7d). The loss of DNAme was most profound in regions associated with active enhancers (Fig. 3b). In contrast, widespread reduction of H3K27me3 was observed across most investigated regions (Fig. 3c) accompanied by the gain of H3K4me3 at regions designated as active TSS and bivalent TSS/enhancer (Fig. 3d) in samples treated with UNC1999 alone or in combination with 5-azacytidine. Notably, the regions that lost H3K27me3 upon EZH2 inhibition were also enriched for H3K27me3 in MM patients compared to NPCs (Fig. 3e).

**Fig. 4:**
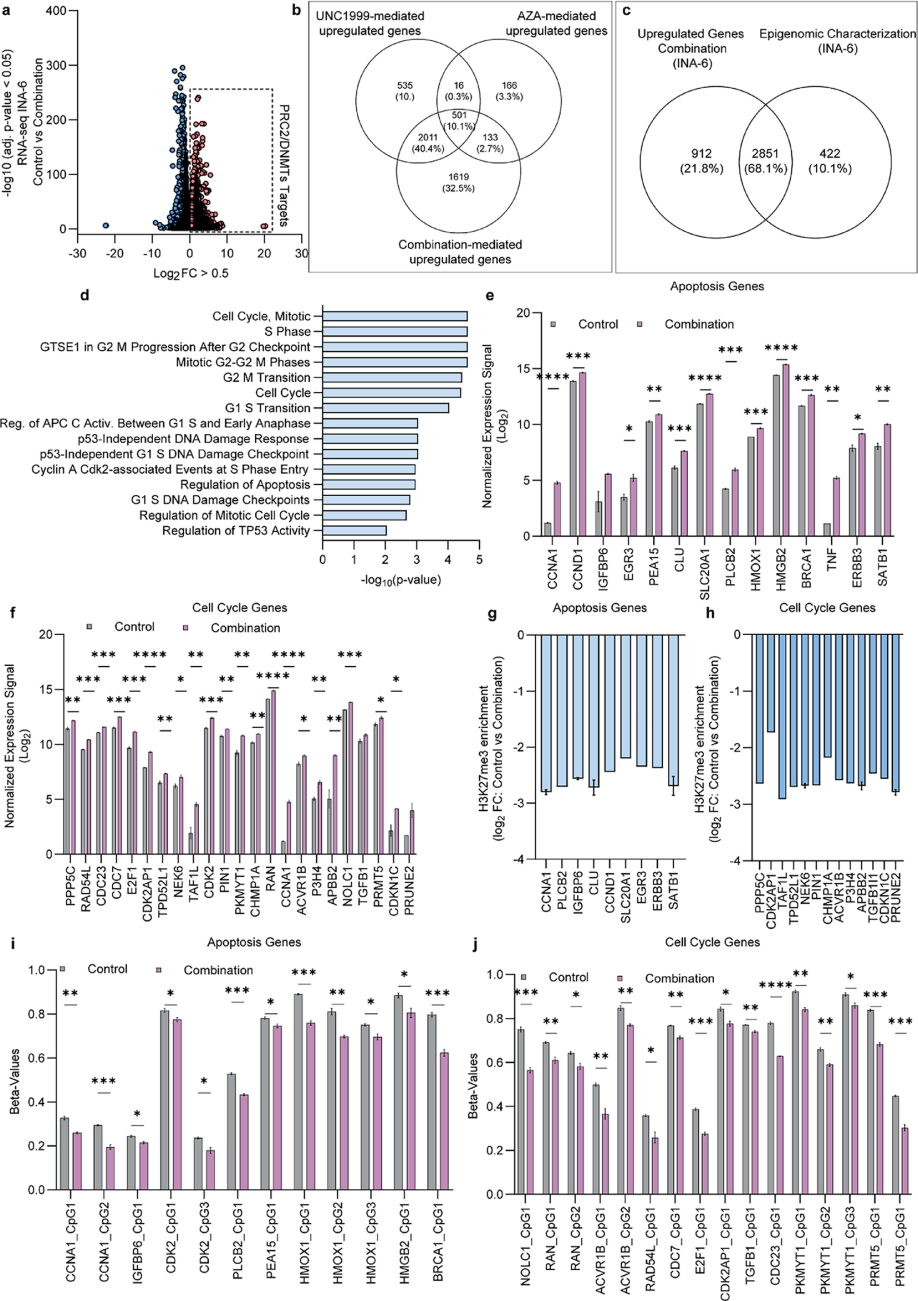
Combinatorial treatment with UNC1999 and 5-azacytidine uniquely promotes the activation of genes associated with apoptosis and cell cycling. **(a)** RNA-seq data for the INA-6 MM cell line treated for a total of 9 days with the UNC1999/5-azacytidine (AZA) combination, with upregulated genes being classified as PRC2/DNMTs targets (blue circles indicate gene downregulation and light red circles indicate gene upregulation). Differential expression was determined by p-value < 0.05 and Log_2_ fold-change > 0.5. Statistical analysis was performed with Wald test and corrected for multiple testing with Benjamini-Hochberg False Discovery Rate (FDR) adjustment (n_vehicle_=3) (n_combination_=3). **(b)** Overlap of genes gaining expression in the monotherapies individually and in the UNC1999/AZA combination. **(c)** Overlap between upregulated genes in the combination therapy with the generated epigenomic map of UNC1999/5-azacytidine targets in the INA-6 MM cell line. **(d)** Bar graph of enriched Reactome Pathways 2024 based on upregulated DEGs (*n* = 2631) unique to the UNC1999/AZA combination treatment, generated using Enrichr. Values as -log_10_ Adj. P-value. **(e)** Upregulated genes associated with apoptosis in the INA-6 MM cells in response to the combinatorial treatment with UNC1999 and 5-azacytidine. Statistical analysis was performed with multiple unpaired t-test, corrected for multiple testing with Holm-Šídák method. Values presented with SEM (n_vehicle_=3) (n_combination_=3). **(f)** Upregulated genes associated with cell cycle in the INA-6 MM cells in response to the combinatorial treatment with UNC1999 and 5-azacytidine. Statistical analysis was performed with multiple unpaired t-test, corrected for multiple testing with Holm-Šídák method. Values presented with SEM (n_vehicle_=3) (n_combination_=3). **(g)** H3K27me3 enrichment of genes associated with apoptosis in the MM cell line INA-6 post-combinatorial treatment. **(h)** H3K27me3 enrichment of genes associated with cell cycle in the MM cell line INA-6 post-combinatorial treatment (n_vehicle_=3) (n_combination_=3). **(i)** DNA methylation levels of genes associated with apoptosis in the MM cell line INA-6 post-combinatorial treatment. Statistical analysis was performed with multiple unpaired t-test, corrected for multiple testing with Holm-Šídák method. Values presented with SEM (n_vehicle_=3) (n_combination_=3). **(j)** DNA methylation levels of genes associated with cell cycle in the MM cell line INA-6 post-combinatorial treatment. Statistical analysis was performed with multiple unpaired t-test, corrected for multiple testing with Holm-Šídák method. Values presented with SEM (n_vehicle_=3) (n_combination_=3), *p < 0.05, **p < 0.01, ***p < 0.001, ****p < 0.0001.

We next performed RNA-seq on the samples treated with either monotherapy or the combination of EZH2 and DNAme inhibitors and identified profound changes in RNA expression (Fig. 4a). Among the upregulated genes, 2011 genes were induced by EZH2 inhibition by UNC1999 either alone or in combination with 5-azacytidine. A distinct set of 133 genes was upregulated by 5-azacytidine monotherapy or in combination with UNC1999, but not by UNC1999 alone. In addition, 1619 genes were uniquely upregulated following the combinatorial treatment (Fig. 4b). To correlate the epigenomic changes with transcriptional activation, we generated an epigenetic map of 3271 target genes affected by the combined treatment, defined as genes that lose H3K27me3 and/or lose DNAme, and/or gain H3K4me3 (Supplementary Table II). Of these, 2851 genes (2631 after ensemble ID conversion) showed increased transcription upon the combinatorial treatment in conjunction with either loss of H3K27me3 or DNAme and/or gain of H3K4me3 (Fig. 4c) (Supplementary Table III). Pathway enrichment analysis of the 2631 differentially expressed genes (DEGs) using Reactome Pathway 2024 Atlas in Enrichr^34^ demonstrated significant enrichment for pathways associated with cell cycling and apoptosis (Fig. 4d). Interestingly, dual inhibition of EZH2 and DNMTs activated a set of apoptosis and cell cycle genes that were not induced by either of the monotherapies (Fig. 4e-f). The upregulation of these genes was directly related to loss of H3K27me3 across the promoter/TSS and gene body (within TSS-2.5Kb to 3’UTR) (Fig. 4g-h) or with decreased DNAme over the gene protomer/TSS (within TSS-2.5Kb to TSS + 1Kb) (Fig. 4i-j).

**Fig. 5:**
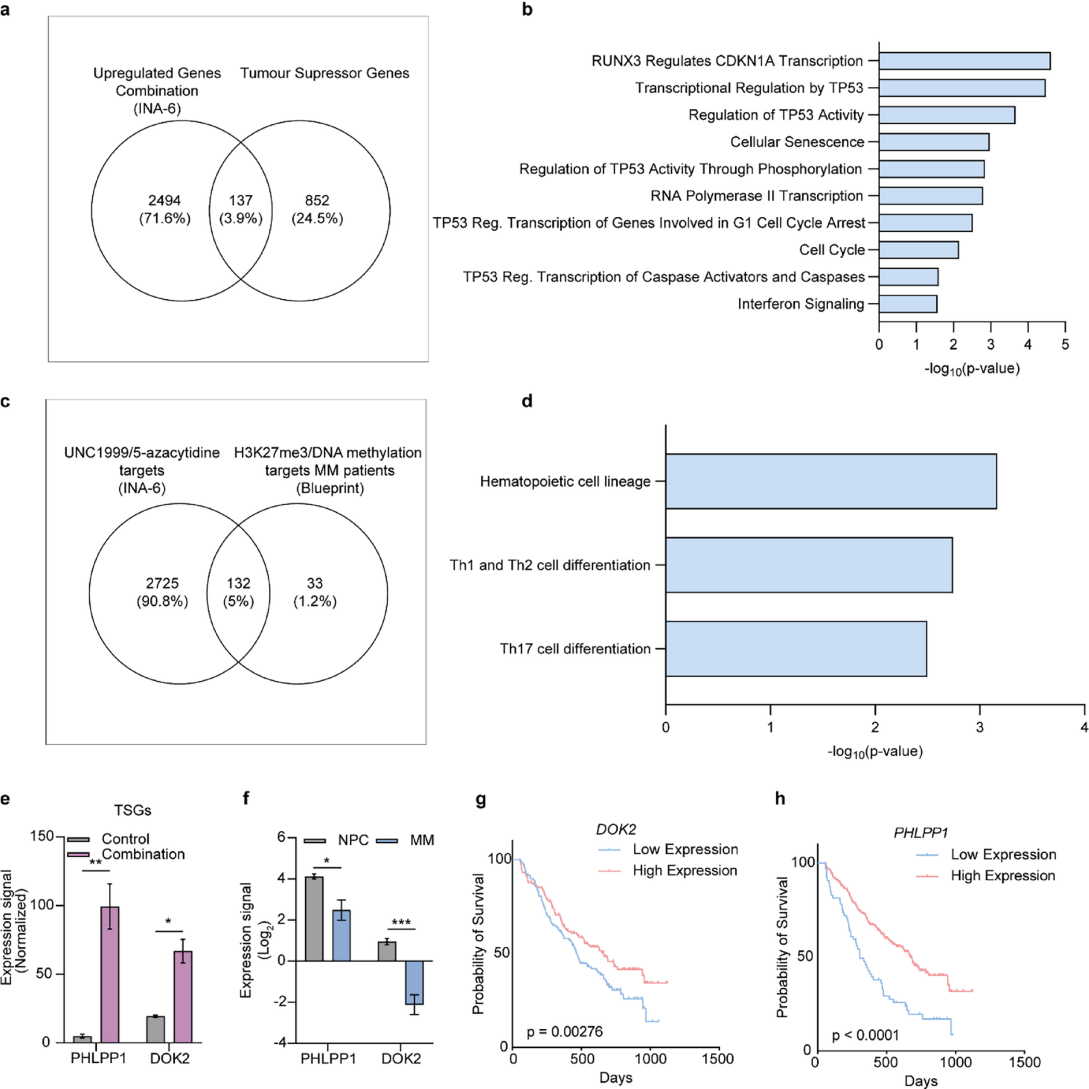
Downregulation of the identified tumour suppressor genes *DOK2* and *PHLPP1* is associated with poor prognosis in MM patients. **(a)** Venn diagram of the overlap between upregulated genes in the MM cell line INA-6 in response to dual EZH2/DNMTs inhibition and previously identified tumour suppressor genes (TSGs) in cancer. **(b)** Bar graph of enriched Reactome Pathways 2024 based on the TSGs unique to the combination treatment (n = 137), generated using Enrichr. Values as -log_10_ Adj. P-value. **(c)** Venn diagram of the overlap between genes bearing H3K27me3 and/or marked by DNA methylation in MM patients compared with normal plasma cells (NPCs), and genes showing loss of H3K27me3 and/or DNA methylation and increased gene expression following dual inhibition of EZH2 and DNMTs in the INA-6 MM cell line. **(d)** Bar graph of enriched KEGG 2021 Human pathways based on the genes (*n* = 132) unique to the combination treatment and gaining H3K27me3/DNA methylation in MM patients compared with NPCs, generated using Enrichr. Values as -log_10_ Adj. P-value. **(e)** RNA-seq data in the INA-6 MM cells after dual EZH2/DNMTs inhibitor treatment for the TSGs *DOK2* and *PHLPP1.* Statistical analysis was performed with multiple unpaired t-test. Values presented with SEM (n_vehicle_=3) (n_combination_=3). **(f)** RNA-seq data for the TSGs *DOK2* and *PHLPP1 *in NPCs (*n* = 3) and MM patients (*n* = 3). Statistical analysis was performed with multiple unpaired t-test, corrected for multiple testing with Holm-Šídák method. Values presented with SEM. Summary of primary samples from the Blueprint Consortium can be found in supplementary Table I. **(g-h)** Normalized (MAS5) *DOK2* and *PHLPP1* expression-based survival data from BM collected CD138^+^ MM patient cells (GSE9782, *n* = 264). Kaplan-Meier curves for overall survival of MM patients with *DOK2* and *PHPLPP1* expression. Statistical test: Log-rank (Mantel-Cox) test. *p < 0.05, **p < 0.01, ***p < 0.001, ****p < 0.0001.

In addition, we observed upregulation of 137 tumour suppressor genes (TSGs) upon the combinatorial treatment with UNC1999 and 5-azacytidine (Fig. 5a) (Supplementary table IV). Reactome pathway analysis of these genes (*n* = 137) revealed enrichment in pathways associated with TP53 activity, cell cycle regulation and interferon signalling (Fig. 5b). Next, we integrated our epigenomic map of UNC1999/5-azacytidine targets (i.e., genes that lose H3K27me3 and/or lose DNAme, and/or gain H3K4me3) with the DNAme, ChIP-seq and RNA-seq data from MM patient samples (Blueprint). We identified 132 genes marked by H3K27me3 and/or by DNAme in MM patient samples compared to NPCs (Fig. 5c). These genes overlapped with regions that showed epigenetic reprogramming following dual treatment and were enriched in the hematopoietic cell lineage pathway (Fig. 5d). (Supplementary Table V). Finally, common among these two lists were the tumour suppressor genes *DOK2* and *PHLPP1* that demonstrated increased gene expression in response to dual inhibition of EZH2 and DNMTs in MM cells (Fig. 5e) and lower expression in MM patient plasma cells compared to NPCs (Fig. 5f). Interestingly, loss of *DOK2* and *PHLPP1* expression was found to be associated with poor prognosis in MM patients (Fig. 5g-h).

**Fig. 6:**
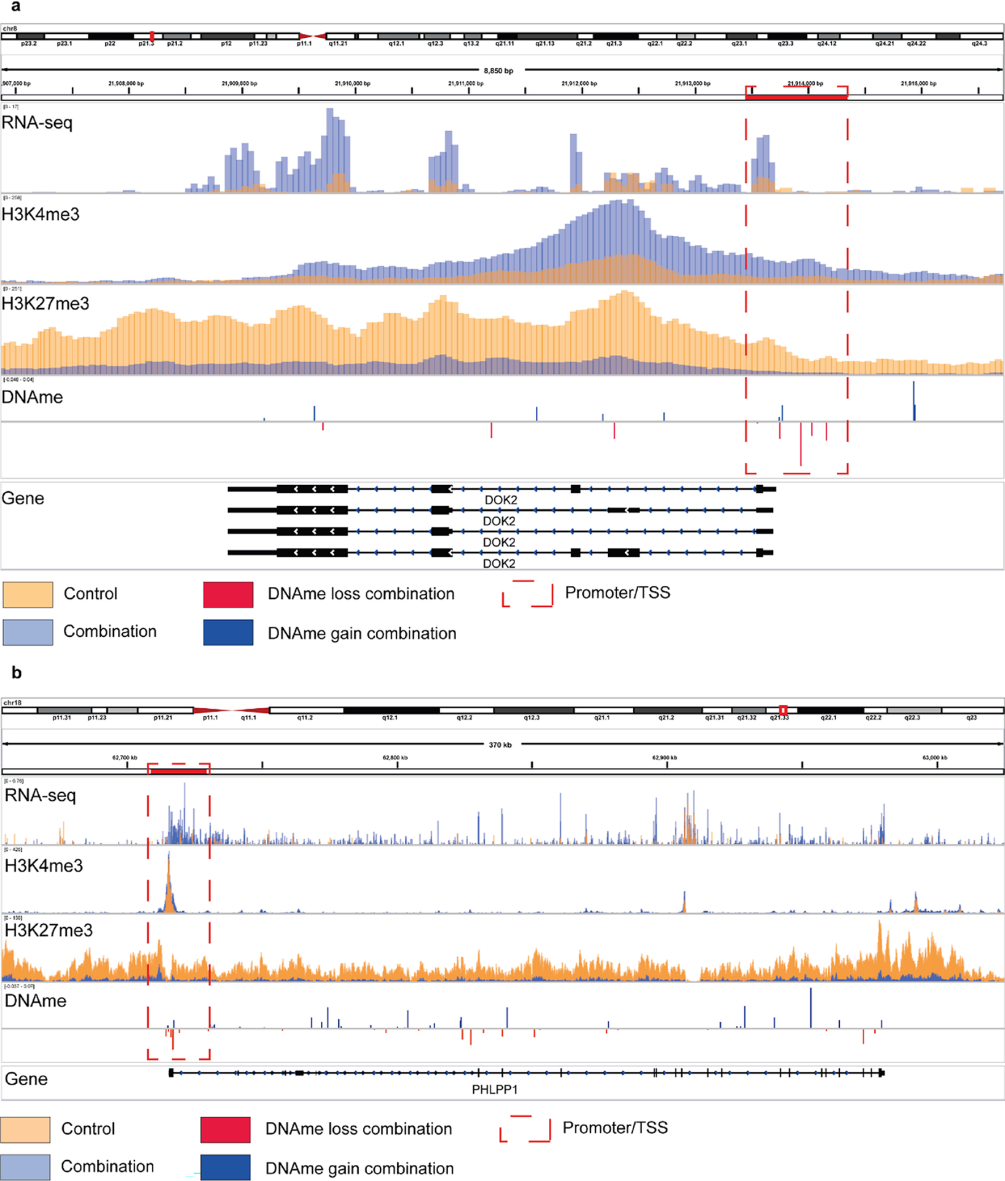
Landscape representation of RNA-seq, ChIP-seq and Illumina Infinium MethylationEPIC arrays data from the INA-6 MM cell line treated with the combination of UNC1999 and 5-azacytidine showing gain of expression, loss of H3K27me3, gain of H3K4me3 and loss of DNA methylation in the tumour suppressor genes **(a)** *DOK2* and **(b)** *PHLPP1*. IGV coverage plot showing normalised read count of RNA-Seq and ChIP-Seq for control (orange) and combination (blue) samples. The DNA methylation (DNAme) difference between combination and control is also shown where a loss/gain of DNAme in the combination treatment is marked in red/blue. Red squares indicate promoter/transcription start sites. All experiments were conducted in minimum three independent biological replicates.

Both *DOK2* and *PHLPP1* genes demonstrated significantly increased transcription, while simultaneously losing H3K27me3 enrichment and gaining, although not significant, H3K4me3 enrichment, around the promoter/TSS as a result of the combinatorial treatment with UNC1999 and 5-azacytidine (Fig. 6a-b).

### Combinatorial treatment with UNC1999 and 5-azacytidine blocks proliferation and induces apoptosis in MM

**Fig. 7:**
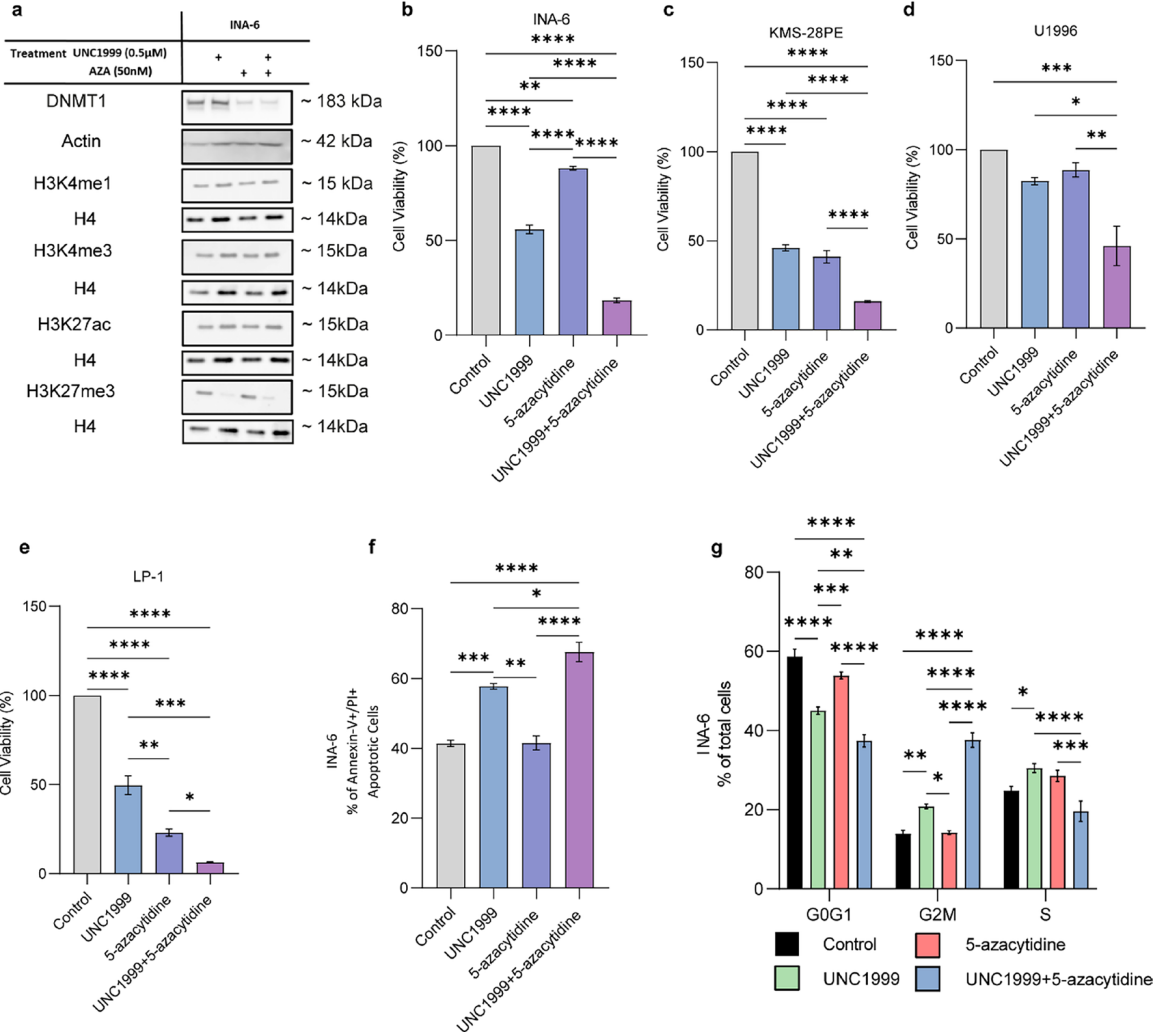
Combined inhibition of DNMTs and EZH2 reduces cell viability, induces accumulation of cells in the G2/M phase of the cell cycle, and promotes apoptosis. (**a**) Western blot analysis of protein and histone marks availability upon treatment with UNC1999 and 5-azacytidine (AZA) alone or in combination for the total of 9 days in the INA-6 MM cell line. Actin and total histone H4 (H4) were used as loading controls for DNMT1 and the presented chromatin modifications, accordingly. Blots are representative from three independent biological experiments. Corresponding uncropped western blot images can be found in supplementary Fig. 13. **(b-e)** Viability analysis by the resazurin reduction method in the corresponding MM cell lines upon treatment with UNC1999 and 5-azacytidine alone or in combination for the total of 9 days. Statistical analysis was performed with one-way Anova. Values presented with SEM (n_vehicle_=3) (n_combination_=3). **(f)** Induction of apoptosis in the INA-6 MM cell line upon treatment with UNC1999 and 5-azacytidine alone or in combination for the total of 9 days. Corresponding gating can be found in supplementary Fig. 11. Statistical analysis was performed with one-way Anova. Values presented with SEM (n_vehicle_=3) (n_combination_=3). **(g)** Cell cycle analysis in the INA-6 MM cell line upon treatment with UNC1999 and 5-azacytidine alone or in combination for the total of 9 days. Corresponding gating can be found in supplementary Fig. 12. Statistical analysis was performed with two-way Anova. Values presented with SEM (n_vehicle_=3) (n_combination_=3). *p < 0.05, **p < 0.01, ***p < 0.001, ****p < 0.0001.

Having demonstrated a direct interplay between DNMT1 and EZH2, we next sought to determine the phenotypic consequences of their combined inhibition. Consistent with our ChIP-seq and DNAme findings, global DNMT1 protein levels were reduced following 5-azacytidine alone or in combination with UNC1999 (Fig. 7a). Similarly, UNC1999 reduced the protein levels of H3K27me3 either in combination or as a monotherapy (Fig. 7a). The global levels of the histone marks H3K27ac, H3K4me1 and H3K4me3 remained largely unchanged after either treatment (Fig. 7a).

Notably, co-inhibition of EZH2 and DNMTs significantly reduced viability compared to single-agent treatment across a panel of MM cell lines (Fig. 7b-e) and demonstrated a positive Bliss score, suggesting that the effect of the drug combination surpasses the expected effect, indicating synergy (Supplementary Fig. 8). We next observed that the effects on cell viability could be explained by a higher degree of apoptosis after combinatorial treatment with UNC1999 and 5-azacytidine compared to the single-agent treatment (Fig. 7f). Furthermore, cell cycle analysis revealed accumulation of cells in the G2/M phase (Fig. 7g).

Altogether, these results show that combined inhibition of EZH2 and DNMTs promotes apoptosis and impairs proliferation in MM cells.

## Discussion

Multiple myeloma (MM) is a highly heterogeneous disease characterised by diverse genomic aberrations, gene expression profiles, and clinical outcomes. However, we and others have demonstrated that EZH2 overexpression is a common feature among MM patients and is associated with shorter survival, independent of established prognostic factors such as the International Staging System (ISS), GEP70 risk and cytogenetic classification^6,13^. Additionally, EZH2 was recently identified to be further significantly upregulated in extramedullary MM (EMM), an ultimately aggressive and hard to treat form of the disease^35^. Moreover, we previously demonstrated that MM cell lines exhibit differential sensitivity to the EZH2-specific inhibitor UNC1999^32^, underscoring the need for novel combinatorial treatment strategies to overcome resistance and improve therapeutic efficacy.

Here, we present a comprehensive map of regulatory regions across the genome of normal plasma cells (NPCs), defined by combinatorial patterns of histone modifications and chromatin accessibility. By comparing MM patients with NPCs, we uncovered profound alterations in the malignant onco-epigenome, with extensive reorganization of all examined histone marks. We found redistribution of H3K27me3 selectively targeted to specific loci and widespread H3K27ac enrichment in active TSS and TSS flanking regions, likely contributing to the silencing and activation of specific gene subsets. Previously, the genome-wide enrichment of H3K27ac and increased chromatin accessibility in primary MM samples have been attributed to decomposition of heterochromatin and linked to activation of numerous enhancers and subsequent signalling pathways that underly myelomagenesis^8,10,11^. Here, increased H3K27me3 accompanied by reduced transcriptional activity was linked to dysregulation of IL-2-STAT5 signalling, a pathway critical for normal plasma cell differentiation^36^. Similarly, enhancer regions, exhibited localized and selective increases in H3K27me3, suggesting targeted epigenetic silencing at these loci. Recently, the tumour-suppressive H3K27 demethylase KDM6A was suggested to guide increased H3K27ac at enhancers, whereas its frequent loss in MM could be attributed to increase of H3K27me3 levels and silencing of immune response genes^37^.

Consistent with findings in other cancers, we found overall DNA hypomethylation in MM patient samples. During the transition from the pre-malignant stage of MGUS to MM, global hypomethylation occurs, however, this is accompanied by gene-specific hypermethylation at promoter regions of tumour suppressor genes^4,38^. Although mutations in DNMT genes have been identified in MM and are considered early events in disease pathogenesis, these alterations are relatively rare and do not correlate with patient prognosis^39^. In the absence of such mutations, previous studies have reported increased DNMT1 and DNMT3A protein levels in MM cells^16^. In line with previous findings, we demonstrated that TSS and enhancer regions present with a higher median DNA methylation in MM patients, consistent with the earlier reports of hypermethylation at immature B cells-specific enhancers in MM^5^. In acute myeloid leukaemia (AML), treatment with the DNA demethylating agent 5-azacytidine has shown clinical benefit and has been effective in overcoming resistance to conventional chemotherapeutics^40^. In contrast, most preclinical studies in MM have employed high doses of 5-azacytidine, which are likely not clinically feasible, and the precise mechanism of action of this drug is still poorly understood. Our data demonstrated that prolonged treatment with low doses of 5-azacytidine effectively inhibits DNMTs and induces global DNA demethylation. This approach led to the demethylation and subsequent activation of apoptosis-related genes.

The PRC2 complex has been thought to interact with other epigenetic silencers, including HDACs, DNMTs and lncRNAs^41^. However, the relationship between EZH2 and DNMTs remains largely unclear with studies reporting contradictory results^20,42^. Here, we used PLA and co-IP to demonstrate a direct physical interaction between DNMT1 and EZH2, which prompted us to explore whether dual inhibition of these enzymes could exert anti-MM effects.

Numerous reports have focused on the preclinical evaluation of epigenetic therapies in cancer, particularly in haematological malignancies. However, comprehensive genome-wide analyses of the effects of these treatments, either as monotherapies or in combination remain limited. In order to evaluate the consequences of combined EZH2 and DNMT inhibition on the epigenome of MM, we utilised ChIP-seq combined with Illumina Infinium MethylationEPIC arrays and RNA-seq. Our data revealed a reduction of DNA methylation in active enhancer regions, while regions classified as Polycomb and bivalent TSS/enhancer showed a reduction of H3K27me3. Interestingly, we observed a gain in H3K4me3 in active TSS and bivalent TSS/enhancer clusters, suggesting that the combinatorial treatment may replace silencing marks with such associated with active transcription. Importantly, these regions were more enriched in H3K27me3 in MM patient samples than in NPCs, underscoring the potential clinical relevance of their reactivation through dual EZH2 and DNMTs inhibition.

Next, we sought to identify genes that may be critical for the observed reduction of cell viability following the combinatorial treatment, beyond the effects of either monotherapy. Specifically, we aimed to pinpoint genes that were reactivated by the combination therapy. To this end, we focused on genes that, after dual inhibition: (1) lose H3K27me3 and/or DNA methylation within TSS regions (2) and/or gain/maintain H3K4me3 enrichment. A total of 2851 genes fulfilled these criteria.

Interestingly, a subset of genes were key regulators of cell fate including control of apoptosis and the cell cycle. Among them, 132 genes overlapped with those marked by H3K27me3 and downregulated in MM patients when compared with NPCs. Notably, this group included *PHLPP1* and *DOK2.* DOK2 is a member of the phosphotyrosine adaptor family, known to act as substrates of protein tyrosine kinases and to regulate the cell cycle progression, transformation and differentiation in hematopoietic stem and progenitor cells^43^. Loss of *DOK2* expression mediated by DNA methylation is reported as a biomarker for poor prognosis in AML^44^. PHLPP1 is a negative regulator of the Akt kinase pathway, and loss of *PHLPP1* expression in CLL promotes activation of Akt and ERK kinases, allowing for increased proliferation^45^. In MM, we found that loss of either *PHLPP1* or *DOK2* was associated with a poor prognosis, further validating the potential clinical value of reactivating these genes through epigenetic therapy.

A limitation of our study is that in vitro validation was performed by comprehensive genome-wide analysis in the INA-6 cell line and phenotypically in the INA-6, LP-1, KMS28-PE and U1996 MM cell lines. However, the study hypothesis is grounded in functional, clinically relevant findings observed in MM patients, independent of genetic background. These findings were subsequently validated in vitro and further supported by comparison with patient-derived data to reinforce their clinical relevance. Additional experiments validating the phenotypic role of DOK2 and PHLPP1 would also provide insight into their direct relationship to MM pathogenesis, however in our study they represent functional validation of the large-scale epigenetic alterations occurring as a result of the combinatorial treatment. Although in vivo experiments would further strengthen the potential for clinical implementation, they are not required to support the core conclusions regarding the epigenetic reprogramming established in this study.

In summary, we generated a genome-wide map of epigenomic alterations in MM patients and annotated functional clusters that acquire H3K27me3 and/or DNA methylation. We demonstrated an epigenomic reconfiguration following inhibition of DNA methylation and H3K27me3, accompanied by altered expression of genes involved in MM tumour growth and survival. We could demonstrate a physical interaction between DNMT1 and EZH2 in MM cells, suggesting functional interplay between these two epigenetic silencers. Finally, we demonstrated that combinatorial inhibition of EZH2 and DNMTs reduces cell viability by activating genes associated with apoptosis and cell cycling regulation.

## Methods

### Cell culture

The MM cell lines INA-6^46^ (obtained from the Leibniz Institute DSMZ), KMS-28PE^47^ (obtained from the JCRB Cell Bank), LP-1^48^ (obtained from the Leibniz Institute DSMZ) and U1996^49^ were cultured in RPMI-1640 AQmediaTM media (Gibco; Thermo Fisher Scientific, Inc., Waltham, MA, USA; cat. no 31870025) supplemented with 10% heat-inactivated FBS (Gibco; cat. no 10270106), 1), 1% GlutaMAX^TM^-I (Gibco; cat. no 35050038) and antibiotics (penicillin 100U/ml and streptomycin 50 mg/mL) (Gibco; cat. no 15140122) at 37 °C in a humidified 5% CO_2_ in-air atmosphere incubator. The INA-6 and U1996 cell lines were supplemented with Interleukin-6 (IL-6). Exponentially growing cells were seeded at 100.000 cells/mL and incubated overnight before addition of reagents. Every 72 h, cells were split in equal numbers and re-plated. Prior to experimental procedures, all cell lines were tested for potential mycoplasma infections using MycoAlert Mycoplasma Detection Kit (Lonza; Basel, Switzerland; cat. no LT07118).

### Reagents and treatment

The demethylating agent 5-azacytidine was purchased from Abcam (Abcam, Cambridge, UK; cat. no ab142744) and added (12.5nM-200nM) daily during the entire duration of the experiments. UNC1999 kindly provided by Anqi Ma and Jian Jin and was dissolved according to previously published procedures and refreshed every 72 h^22^. For the combinatorial treatment the MM cell lines were pre-treated with 5-azacytidine for the duration of 3 days after which UNC1999 was added and the experiment continued until the indicated time point.

### Cell viability assay

The MM cell lines used in this study were treated with UNC1999, 5-azacytidine or the combination of both for the detailed durations. For the combinatorial treatment the MM cell lines were pre-treated with 5-azacytidine for the duration of 3 days after which UNC1999 was added and the experiment continued until the indicated time point. DMSO was used as a control vehicle treatment. At each indicated time-point, cells were transferred to 96-well flat-bottom plates and viability was assessed using the resazurin reduction method using AlamarBlue™ Cell Viability reagent (Sigma Aldrich, MI, USA, cat. no R7017) as previously described^12^. Combinatorial index was calculated as previously described^50^.

### Cell cycle and apoptosis analysis

Cell cycle analysis was performed by using BrdU incorporation as per the manufacturer’s instructions with 1 h incubation of BrdU and cell culture (BD Pharmingen, CA, USA: ca.t no 559619). Apoptosis was measured by using the FACS Annexin V-FITC apoptosis detection kit (R&D Systems, MN US: cat. no 4830) and apoptotic cells were presented as percentage of Annexin V-positive/PI-negative cells. Analysis was performed by flow cytometry using the BD LSRFortessa™ (BD Biosciences, NJ, USA).

### Co-immunoprecipitation (Co-IP)

One milligram of protein was used for Co-IP using the Pierce Classic IP Kit (Thermo Fisher Scientific, Waltham, MA, USA; cat. no26146). In brief, cells were harvested and protein extracted using the IP lysis/wash buffer. Protease inhibitors were added to IP lysis/wash buffer and the complete buffer was used at 400 µl/10 × 10^6^ cells. Ten micrograms of primary anti-EZH2 (Millipore, MA, USA: cat. no 17–662) and anti-IgG (Millipore, MA, USA: cat. no12-371B) control antibody was used for protein pull-down. The Novex Sharp pre-stained Protein Ladder (Thermo Fisher Scientific, Waltham, MA, USA; cat. no LC5800) and MagicMark XP Western Protein Standard (Thermo Fisher Scientific, Waltham, MA, USA; cat. no LC5602) were used as size ladder markers. Sample-Buffer Elution was used to elute sample for downstream DNMT1 western blot analysis as previously described below using anti-DNMT1 (Abcam, Cambridge, UK; cat. no 19905).

### RNA extraction

RNA extraction was performed with RNeasy Micro and/or Mini Kit (Qiagen, Netherlands: cat. no 74004/74104) as described by the manufacturer.

### Proximity ligation assay (PLA)

In situ PLA was performed with the Duolink^®^ In Situ Red Starter Kit Mouse/Rabbit (Sigma Aldrich, MO, USA) according to the manufacturer’s instructions. In brief, PFA-fixed cells on glass slides were encircled with ImmEdge Hydrophobic Barrier PAP Pen (Vector Laboratories, CA, USA) to ensure the reaction mixes covered the cells. The cells were then permeabilized with 1x TBS (Thermo Fisher Scientific, Waltham, MA, USA) with 0.2% v/v Triton X-100 (Sigma-Aldrich, MO, USA) for 15 min, followed by 2 min wash with 1x TBS. Blocking was performed for 1 h with Duolink blocking buffer, and afterwards the cells were incubated with a mixture of primary antibodies against the proteins of interest (anti-EZH2: Millipore, MA, USA: cat. no 17–662 or anti-DNMT1: Abcam, Cambridge, UK; cat. no #19905) raised in different hosts (mouse and rabbit), or with either one of these antibodies (to serve as omitting controls). Primary antibodies were incubated overnight at 37 °C, followed by incubation with a mix of the Duolink^®^ In Situ PLA^®^ Probe Anti-Rabbit PLUS, Affinity purified Donkey anti-Rabbit IgG (H + L) and Duolink^®^ In Situ PLA^®^ Probe Anti-Mouse MINUS, Affinity purified Donkey anti-Mouse IgG (H + L) in the concentrations recommended by the supplier. Next followed hybridization and ligation with the Duolink Ligation mix, and finally, rolling circle amplification and signal detection were performed using the Duolink^®^ In Situ Detection Reagent Red, which results in rolling circle amplification products that are fluorescently labelled in TexasRed. Nuclei were labelled with Hoechst33342 (1:250). The slides were then mounted with SlowFade Gold antifade reagent (Thermo Fisher Scientific, Waltham, MA, USA) and images were acquired with a Zeiss AxioImager M2 using a Zeiss Plan-Apochromat 40x NA 1,4 oil objective.

### RNA-seq, library Preparation and analysis (INA-6 cell line)

The INA-6 cell line treated with vehicle DMSO, 0.5µM UNC1999, 50nM 5-azacytidine or the combination of both for the total of 9 days. For the combinatorial treatment the cells were pre-treated with 5-azacytidine for the duration of 3 days after which UNC1999 was added and the experiment continued until the indicated time point. Purified RNA was measured using Qubit™ (Thermo Fisher Scientific, Waltham, MA, USA). RNA integrity was then measured and calculated on Tapestation™ (Agilent, CA, USA). ~100 ng of RNA was used for sample library preparation utilizing the TruSeq Stranded Total RNA Gold (Illumina, CA, USA), including Ribo-Zero™ (Illumina, CA, USA) and non-poly-A selection. The samples were sequenced for 50 cycles pair-end on one SP flow cell on the NovaSeq 6000 system (Illumina, CA, USA). All samples were sequenced in biological triplicates.

The RNA-seq analysis was performed using the nf-core^51^ RNASeq pipeline (10.5281/zenodo.3503887) in version 3.4 using default parameters for paired-end sequencing. Differential expression analysis was performed using the DESeq2 workflow as outlined in the Bioconductor workflow presented here: http://master.bioconductor.org/packages/release/workflows/vignettes/rnaseqGene/inst/doc/rnaseqGene.html.

### ChIP-seq, library Preparation and analysis (INA-6 cell line)

ChIP was conducted by utilizing the iDeal ChIP-seq Kit for Histones (Diagenode, Belgium, cat. noC01010051) according to the manufacturer’s instructions. In short, 7 million INA-6 cells treated with vehicle DMSO, 0.5µM UNC1999, 50nM 5-azacytidine or the combination of both for the total of 9 days. For the combinatorial treatment the cells were pre-treated with 5-azacytidine for the duration of 3 days after which UNC1999 was added and the experiment continued until the indicated time point. Cells were cross-linked for 8 min at room temperature in 1% formaldehyde (Thermo Scientific, Waltham, MA, USA: cat. no 28906). Then, 0.1 M of glycine was added to cease the cross-linking reaction. Shearing of chromatin was done in 10 cycles on the PicoBioruptor™ (Diagenode) (30 s on/30 sec off). Samples were then incubated with either anti-H3K27me3 (5.68 µg) (Diagenode, Belgium, cat. no C15410196), anti-H3K27ac (2 µg) (Active Motif, CA, USA: cat. no 39155), anti-H3K4me1 (2 µg) (Abcam, Cambridge, UK; cat. no 8895) or anti-H3K4me3 (2 µg) (Millipore, MA, USA: cat. no 07-473) antibodies.

Libraries were generated on the purified DNA using SMARTer ThruPLEX DNA-seq library preparation kit with fragmentation (Takara Bio, Japan). Cluster generation and 50 cycles paired-end sequencing of the libraries was performed in 1 NovaSeq S2 flowcell using the NovaSeq 6000 system and v1.5 sequencing chemistry (Illumina CA, USA).

The ChIP-Seq fastq files were processed using the nf-core^51^ ChiP-Seq pipeline in version 1.2.2 (doi: 10.5281/zenodo.3240506) and using default parameters for broad (H3K27me3, H3K4me1) and narrow (H3K4me3, H3K27ac) peaks. A custom reference genome consisting of the concatenation of the GRCh38 human and *Drosophila melanogaster* (dm6) reference genome sequences was used. Similarly, a custom blacklist BED file was generated by concatenating GRCh38 and dm6 blacklist regions. Briefly, the nf-core pipeline performed the following steps: trim adapters by Trim Galore!, map reads by BWA, filter reads, report QC measures for read alignment and ChIP, call peaks by MACS2, generate a consensus peak set per mark using BEDTools, count reads in consensus peak set by featureCounts. The chromatin samples from the INA-6 cell line were exposed to four different treatments, namely DMSO, 5-azacytidine, UNC1999 and a combination of 5-azacytidine and UNC1999. A constant small amount of sonicated dm6 chromatin spike-ins (33.25-36ng) was added before immunoprecipitation. This was performed for four batches of replicates. A scaling factor was then computed for each replicate batch using DESeq2 function estimateSizeFactorsForMatrix^52^ using counts of dm6 peaks only. All peak counts were subsequently divided by the dm6-derived batch-specific scaling factor to correct for technical variability in the ChIPSeq experiment. To determine peaks that were statistically significant between DMSO, 5-azacytidine, UNC1999 and a combination of 5-azacytidine and UNC1999, we computed the fold change per batch and then performed a hypothesis test using a two-sided t-test on four values of fold changes, and under the null hypothesis that the mean is zero. Finally, the false discovery rate to account for multiple testing was computed using the Benjamini-Hochberg method. Data was visualised with IGV V.2.11.0^53^.

### Protein extraction and Western blot

Following treatments, MM cell lines were harvested, washed with ice-cold PBS and collected at 1500 rpm for 5 min. For non-histone proteins, total cellular protein was extracted using RIPA extraction buffer (Millipore, #20–188) with freshly added protease inhibitor cocktail. Western blotting protocol was performed as previously described^13^. Histone proteins were extracted using the Episeeker histone extraction kit (Abcam, #113476) following the manufacturer’s procedure. The following antibodies were used for immunoblotting: DNMT1 (Abcam, Cambridge, UK; cat. no 19905), DNMT3A (Santa Cruz, TX, USA: cat. no 65769), actin (Santa Cruz, TX, USA: cat. no 1616), H3K4me1 (Abcam, Cambridge, UK; cat. no 8895), H3K4me3 (Millipore, MA, USA: cat. no 07-473), H3K27ac (Millipore, MA, USA: cat. no 07-360), H3K27me3 (Millipore, MA, USA: cat. no 07-449), total histone H3 (Abcam, Cambridge, UK; cat. no 1791) and total histone H4 (Active Motif, Waterloo, Belgium; cat. no 91296).

### DNA methylation analysis

The INA-6 MM cell line was treated with vehicle DMSO, 0.5µM UNC1999, 50nM 5-azacytidine or the combination of both 0.5µM UNC1999 and 50nM 5-azacytidine for the total of 9 days. For the combinatorial treatment the cells were pre-treated with 5-azacytidine for the duration of 3 days after which UNC1999 was added and the experiment continued until the indicated time point. Global DNA methylation was assessed after bisulfite conversion. Bisulfite conversion was performed using the EZ DNA Methylation™ Kit (Zymo Research, CA, USA: cat. no D5004) with 250 ng of DNA per sample. The bisulfite-converted DNA was eluted in 15 µl according to the manufacturer´s protocol, evaporated to a volume of < 4 µl, and used for methylation analysis by the Illumina Infinium MethylationEPIC array^54,55^. Methylation levels were calculated using Illumina Infinium MethylationEPIC.

Methylation β-values (Human Methylation 450k BeadChip Illumina) for 104 MM bone marrow and 8 tonsil NPC samples were obtained from^5^.

### Genomic data (BLUEPRINT)

We downloaded ChIP-seq, ATAC-seq and RNA-seq human genome build 38 (hg38) data for controls and MM subjects from the BLUEPRINT project of haematopoietic epigenomes after approval of our application for data access (Supplementary Table I). We performed peak calling from the bam files for ATAC-seq using MACS2 with “-f BAMPE -g 3049315783 -q 0.05 --nomodel --shift − 100 --extsize 200 -B --SPMR --call-summits –bdg”.

### ChromHMM on data from primary samples

We annotated the genome of control subjects applying the ChromHMM^56^ tool on ChIP-seq (histone modifications) and ATAC-seq data (Supplementary Table I). The BinarizeBed step of ChromHMM was run with the “–peaks” flag, while the LearnModel step was run for 30 states. The predefined region length was kept to its default value of 200 bp. Next, each state was manually assigned a label reflecting the most likely genetic function based on the combination of ChIP-seq and ATAC-seq signals. Then, groups of annotated states were assigned to broader classes based on their functional similarities. We visualized the average signal intensity of each ChromHMM state and compared it between control NPC and MM subjects for ChIP-seq, ATAC-seq and RNA-seq signals using the deepTools functions runComputeMatrix and runPlotHeatmap^57^. Next, we used the peaks called for ChIP-seq and ATAC-seq to identify regulatory regions losing, gaining, or maintaining the intensity of the corresponding genomic signal in MM when compared to control subjects.

### ChromHMM on INA-6 cell line

ChromHMM was run in version 1.23 (http://compbio.mit.edu/ChromHMM/)^56^. The BinarizeBam step was run with default parameters on the ChIP-Seq bam files from the DMSO control treatment. Four replicates were combined to build a single model from this data using the LearnModel step with default parameters for building a model with 5 states. We used all replicates of input DNA as controls in the model building.

### Differential expression of genomic regions (primary samples)

We used kallisto^58^ to perform alignment-free quantification of the mRNA expression of the genomic regions identified by ChromHMM. We first extracted the human genome build hg38 DNA sequence for each genomic region using bedtools’ getfasta function on hg38 and then constructed the index for kallisto. Next, we ran kallisto with “--bias -b 30 for paired-end reads and --bias -b 30 --single -l 200 -s 20” for single-end reads. The differential expression analysis was performed using sleuth^59^.

### Differential methylation analysis

We first lifted the coordinates from the Human Methylation 450k BeadChip (Illumina, CA, USA) and the Illumina Infinium MethylationEPIC (Illumina, CA, USA) arrays to hg38 using the files from^60^. We used ChAMP^61^ to perform the differential analysis on control and MM subjects. We applied the same pipeline to perform a pair-wise differential analysis for methylation sites on INA-6 treated with vehicle, UNC1999, 5-azacytidine and the combination of both. Similarly, after identifying the shared methylation sites between the Human Methylation 450k BeadChip and the Illumina Infinium MethylationEPIC arrays, we performed a differential analysis between methylation levels of MM subjects and the INA-6 cells treated with vehicle, UNC1999, 5-azacytidine and the combination of both, methylation levels.

### Drug synergy evaluation

Bliss independence model and Combinatorial Index (CI) were used to evaluate combinatorial effect of UNC1999 and 5-azacytidine, with positive values indicating synergy effect of both drugs. Bliss scores were calculated utilizing SynergyFinder+.

### In silico analysis

#### Patient survival data analysis

Patient survival data was generated at genomicscape.com. We utilised the Mulligan myeloma cohort (GSE9782)^62^. All data was analysed and visualized with Graphpad Prism V.9.1.0.

#### Blueprint data analysis

Data from MM samples, normal plasma cells and the human genome build (hg38) was collected from the Blueprint Consortium of hematopoietic epigenomes. Differential analysis of RNA-seq, ATAC-seq and ChIP-seq data was processed using the nf-core pipelines as previously described^51^. All data was visualised and processed in GraphPad Prism V.9.1.0.

#### Gene set enrichment analysis

Gene set enrichment analysis (GSEA) was conducted utilizing GSEA V.4.0.3, using the hallmarks for cancer dataset.

#### Cell profiler

PLA images were analysed in CellProfiler V.3.1.8^63^ and protein-protein interactions were calculated in Fiji ImageJ^64^ with the macro^65^.

### Statistical analysis

Test for normality was done by collective assessment of D’Agostino & Pearson-, Anderson-Darling-, Shapiro-Wilk- and Kolmogorov-Smirnov tests. The statistical analysis used for analysing each figure has been specified in the corresponding figure legend. All data was processed in GraphPad Prism V.10.3.1. GSEA was used to identify pathways based off genes that were differentially expressed.

## Supplementary Information

Below is the link to the electronic supplementary material.

Supplementary Material 1

## Data Availability

All newly generated data is deposited in the ArrayExpress collection in BioStudies under the accession numbers, E-MTAB-13136 (INA-6 RNA-seq), E-MTAB-14627 (INA-6 ChIP-seq) and E-MTAB-14582 (INA-6 Illumina Infinium MethylationEPIC Array).
