# Supplementary material for: Combinatorial DNMTs and EZH2 inhibition reprograms the H3K27me3 and DNAme-mediated onco-epigenome to suppress multiple myeloma proliferation
Source: Sci Rep. 2025 Aug 27;15:31568. doi: 10.1038/s41598-025-17093-z (PMC12391466; doi:10.1038/s41598-025-17093-z)
Supplement: Supplementary file 1 — Supplementary Material 1 [file 41598_2025_17093_MOESM1_ESM.docx]

**Combinatorial DNMTs and EZH2 inhibition reprograms the H3K27me3 and DNAme-mediated onco-epigenome to suppress multiple myeloma proliferation**

Alba Atienza Párraga, Patrick Nylund, Klev Diamanti, Berta Garrido-Zabala, Stefania Iliana Tziola, Louella Vasquez, Paul Theodor Pyl, Doroteya Raykova, Aron Skaftason, Anqi Ma, Jian Jin, José Ignacio Martín-Subero, Fredrik Öberg, Elke De Bruyne, Jan Komorowski, Helena Jernberg-Wiklund and Antonia Kalushkova

**Supplementary information**

**Supplementary tables:**

| **Supplementary Table I. Primary samples from the Blueprint Consortium** | | | | | | | |
| --- | --- | --- | --- | --- | --- | --- | --- |
| Tissue | Sex | Cell type/line | Disease | Donor | Experiment | Sample name | Experiment ID |
| tonsil | Female | plasma cell | Control | T14_5 | ChIP Input | S00VKEH1 | ERX940663 |
| tonsil | Female | plasma cell | Control | T14_5 | H3K27ac | S00VKEH1 | ERX712666 |
| tonsil | Female | plasma cell | Control | T14_5 | H3K27me3 | S00VKEH1 | ERX712774 |
| tonsil | Female | plasma cell | Control | T14_5 | H3K36me3 | S00VKEH1 | ERX712660 |
| tonsil | Female | plasma cell | Control | T14_5 | H3K4me1 | S00VKEH1 | ERX712673 |
| tonsil | Female | plasma cell | Control | T14_5 | H3K4me3 | S00VKEH1 | ERX712665 |
| tonsil | Female | plasma cell | Control | T14_5 | H3K9me3 | S00VKEH1 | ERX712693 |
| tonsil | Female | plasma cell | Control | T14_10 | RNA-Seq | S00Y8Q11 |  |
| tonsil | Female | plasma cell | Control | T14_10 | ChIP Input | S00Y8QH1 | ERX941050 |
| tonsil | Female | plasma cell | Control | T14_10 | H3K27ac | S00Y8QH1 | ERX712735 |
| tonsil | Female | plasma cell | Control | T14_10 | H3K27me3 | S00Y8QH1 | ERX712738 |
| tonsil | Female | plasma cell | Control | T14_10 | H3K36me3 | S00Y8QH1 | ERX712736 |
| tonsil | Female | plasma cell | Control | T14_10 | H3K4me1 | S00Y8QH1 | ERX712734 |
| tonsil | Female | plasma cell | Control | T14_10 | H3K4me3 | S00Y8QH1 | ERX712733 |
| tonsil | Female | plasma cell | Control | T14_10 | H3K9me3 | S00Y8QH1 | ERX712737 |
| tonsil | Male | plasma cell | Control | T14_11 | RNA-Seq | S0139T11 |  |
| tonsil | Male | plasma cell | Control | T14_11 | ChIP Input | S0139TH1 | ERX1007401 |
| tonsil | Male | plasma cell | Control | T14_11 | H3K27ac | S0139TH1 | ERX1007382 |
| tonsil | Male | plasma cell | Control | T14_11 | H3K4me1 | S0139TH1 | ERX1007383 |
| tonsil | Male | plasma cell | Control | T14_6 | ChIP Input | S013BPH1 | ERX1122536 |
| tonsil | Male | plasma cell | Control | T14_6 | H3K27me3 | S013BPH1 | ERX1122513 |
| tonsil | Male | plasma cell | Control | T14_6 | H3K9me3 | S013BPH1 | ERX1122531 |
| tonsil | Female | plasma cell | Control | T15_3 | RNA-Seq | S019GS11 |  |
| tonsil | Female | naive B cell | Control | T15_12 | ATAC-seq | S01DHAH1 | ERX1322681 |
| tonsil | Female | plasma cell | Control | T15_12 | ATAC-seq | S01DJ6H1 | ERX1322682 |
| tonsil | Female | germinal center B cell | Control | T15_12 | ATAC-seq | S01DK4H1 | ERX1322680 |
| tonsil | Male | plasma cell | Control | T15_P2 | ChIP Input | S01EBIH1 | ERX1295589 |
| tonsil | Male | plasma cell | Control | T15_P2 | H3K36me3 | S01EBIH1 | ERX1122619 |
| tonsil | Male | plasma cell | Control | T15_P2 | H3K4me3 | S01EBIH1 | ERX1122618 |
| tonsil | Male | naive B cell | Control | T15_18 | ATAC-seq | S01SA171 | ERX1322322 |
| tonsil | Male | plasma cell | Control | T15_18 | ATAC-seq | S01SCY71 | ERX1322324 |
| tonsil | Male | germinal center B cell | Control | T15_18 | ATAC-seq | S01SDW71 | ERX1322323 |
| tonsil | Female | naive B cell | Control | T15_20 | ATAC-seq | S01SEU71 | ERX1322325 |
| tonsil | Unknown | plasma cell | Control | T12-15 | RNA-Seq | T12-15-PC |  |
| tonsil | Unknown | plasma cell | Control | T12-16 | RNA-Seq | T12-16-PC |  |
| tonsil | Unknown | plasma cell | Control | T12-17 | RNA-Seq | T12-17-PC |  |
| tonsil | Unknown | plasma cell | Control | T12-18 | RNA-Seq | T12-18-PC |  |
| bone marrow | Female | plasma cell | Multiple Myeloma | 22965 | RNA-Seq | S00XCM11 |  |
| bone marrow | Female | plasma cell | Multiple Myeloma | 22965 | ATAC-seq | S00XCM71 | ERX1322304 |
| bone marrow | Female | plasma cell | Multiple Myeloma | 22965 | ChIP Input | S00XCMH1 | ERX712742 |
| bone marrow | Female | plasma cell | Multiple Myeloma | 22965 | H3K27ac | S00XCMH1 | ERX712720 |
| bone marrow | Female | plasma cell | Multiple Myeloma | 22965 | H3K27me3 | S00XCMH1 | ERX712712 |
| bone marrow | Female | plasma cell | Multiple Myeloma | 22965 | H3K36me3 | S00XCMH1 | ERX712718 |
| bone marrow | Female | plasma cell | Multiple Myeloma | 22965 | H3K4me1 | S00XCMH1 | ERX712719 |
| bone marrow | Female | plasma cell | Multiple Myeloma | 22965 | H3K4me3 | S00XCMH1 | ERX712716 |
| bone marrow | Female | plasma cell | Multiple Myeloma | 22965 | H3K9me3 | S00XCMH1 | ERX712721 |
| bone marrow | Male | plasma cell | Multiple Myeloma | 15548 | RNA-Seq | S00XDK11 |  |
| bone marrow | Male | plasma cell | Multiple Myeloma | 15548 | ATAC-seq | S00XDK71 | ERX1322305 |
| bone marrow | Male | plasma cell | Multiple Myeloma | 15548 | ChIP Input | S00XDKH1 | ERX941048 |
| bone marrow | Male | plasma cell | Multiple Myeloma | 15548 | H3K27ac | S00XDKH1 | ERX712765 |
| bone marrow | Male | plasma cell | Multiple Myeloma | 15548 | H3K27me3 | S00XDKH1 | ERX712769 |
| bone marrow | Male | plasma cell | Multiple Myeloma | 15548 | H3K36me3 | S00XDKH1 | ERX712767 |
| bone marrow | Male | plasma cell | Multiple Myeloma | 15548 | H3K4me1 | S00XDKH1 | ERX712766 |
| bone marrow | Male | plasma cell | Multiple Myeloma | 15548 | H3K4me3 | S00XDKH1 | ERX712764 |
| bone marrow | Male | plasma cell | Multiple Myeloma | 15548 | H3K9me3 | S00XDKH1 | ERX712768 |
| bone marrow | Female | plasma cell | Multiple Myeloma | 23977 | RNA-Seq | S00XEI11 |  |
| bone marrow | Female | plasma cell | Multiple Myeloma | 23977 | ATAC-seq | S00XEI71 | ERX1322306 |
| bone marrow | Female | plasma cell | Multiple Myeloma | 23977 | ChIP Input | S00XEIH1 | ERX1007376 |
| bone marrow | Female | plasma cell | Multiple Myeloma | 23977 | H3K27ac | S00XEIH1 | ERX1007378 |
| bone marrow | Female | plasma cell | Multiple Myeloma | 23977 | H3K27me3 | S00XEIH1 | ERX1007375 |
| bone marrow | Female | plasma cell | Multiple Myeloma | 23977 | H3K36me3 | S00XEIH1 | ERX1007373 |
| bone marrow | Female | plasma cell | Multiple Myeloma | 23977 | H3K4me1 | S00XEIH1 | ERX1007372 |
| bone marrow | Female | plasma cell | Multiple Myeloma | 23977 | H3K4me3 | S00XEIH1 | ERX1007377 |
| bone marrow | Female | plasma cell | Multiple Myeloma | 23977 | H3K9me3 | S00XEIH1 | ERX1007374 |
| bone marrow | Female | plasma cell | Multiple Myeloma | 54168MM | ATAC-seq | S00XFG71 | ERX1327407 |
| bone marrow | Female | plasma cell | Multiple Myeloma | 54168MM | ChIP Input | S00XFGH1 | ERX1122535 |
| bone marrow | Female | plasma cell | Multiple Myeloma | 54168MM | H3K27ac | S00XFGH1 | ERX1122529 |
| bone marrow | Female | plasma cell | Multiple Myeloma | 54168MM | H3K27me3 | S00XFGH1 | ERX1122512 |
| bone marrow | Female | plasma cell | Multiple Myeloma | 54168MM | H3K36me3 | S00XFGH1 | ERX1122530 |
| bone marrow | Female | plasma cell | Multiple Myeloma | 54168MM | H3K4me1 | S00XFGH1 | ERX1122528 |
| bone marrow | Female | plasma cell | Multiple Myeloma | 54168MM | H3K4me3 | S00XFGH1 | ERX1122527 |
| bone marrow | Female | plasma cell | Multiple Myeloma | 54168MM | H3K9me3 | S00XFGH1 | ERX1122511 |
| Cell Line | Male | U-266 | Multiple Myeloma |  | ChIP Input | U-266_c01 | ERX297449 |
| Cell Line | Male | U-266 | Multiple Myeloma |  | H3K27ac | U-266_c01 | ERX297418 |
| Cell Line | Male | U-266 | Multiple Myeloma |  | H3K27me3 | U-266_c01 | ERX297421 |
| Cell Line | Male | U-266 | Multiple Myeloma |  | H3K36me3 | U-266_c01 | ERX297442 |
| Cell Line | Male | U-266 | Multiple Myeloma |  | H3K4me1 | U-266_c01 | ERX297410 |
| Cell Line | Male | U-266 | Multiple Myeloma |  | H3K4me3 | U-266_c01 | ERX297406 |
| Cell Line | Male | U-266 | Multiple Myeloma |  | H3K9me3 | U-266_c01 | ERX297435 |
| Cell Line | Male | U-266 | Multiple Myeloma |  | DNase-Seq | U-266_d01 | ERX297502 |
| Cell Line | Male | U-266 | Multiple Myeloma |  | RNA-Seq | U-266_r01 |  |

**Supplementary table II. Genes gaining expression and loosing H3K27me3 and/or DNA Methylation and/or gaining H3K4me3.**

Full table can be found in submitted excel document “Supplementary table II”.

**Supplementary table III. Genes overlapping with functional regions in INA-6 cells that gaining expression and loosing H3K27me3 and/or DNA Methylation and/or gaining H3K4me3.**

Full table can be found in submitted excel document “Supplementary table III”.

**Supplementary table IV. Upregulated tumour suppressor genes upon the combinatorial treatment with UNC1999 and 5-azacytidine in INA-6 cells.**

Full table can be found in submitted excel document “Supplementary table IV”.

**Supplementary table V. Genes in MM patients overlapping with functional regions in INA-6 cells that gaining expression and loosing H3K27me3 and/or DNA Methylation and/or gaining H3K4me3.**

Full table can be found in submitted excel document “Supplementary table V”.

**Supplementary figures:**

**­­­­­­­
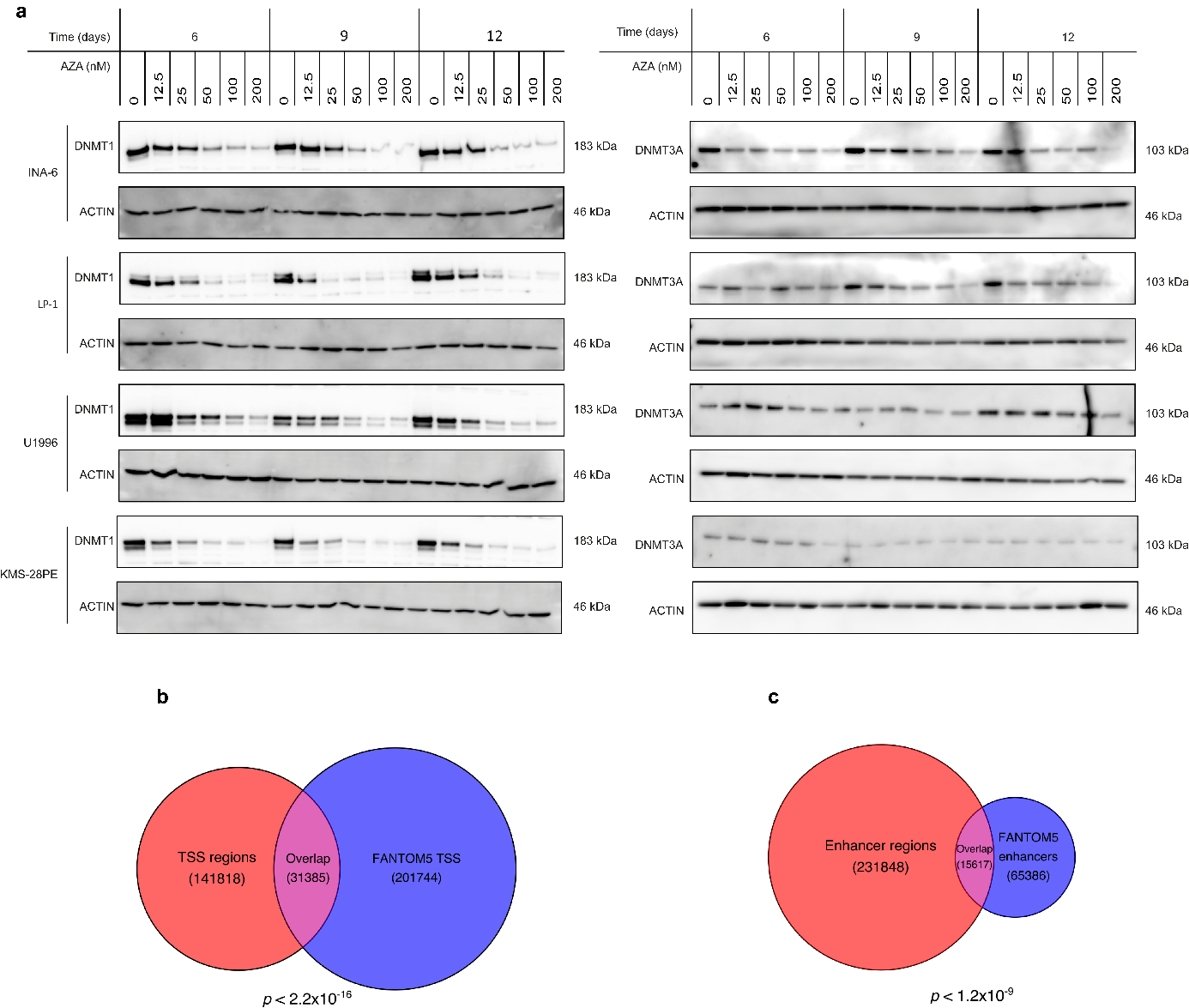
**

**Supplementary figure 1. ChromHMM defined TSS and enhancer regions showed significant overlap with FANTOM5 data. (a)** DNA demethylating treatment using Aza reduces the DNMT1 and DNMT3A protein levels. Actin was used as a loading control. Uncropped western blot images can be found in supplementary figure 14. **(b-c)** Significance of the intersection between the classes of promoters/TSS and enhancers identified by ChromHMM, with TSSs and enhancers identified by FANTOM5. We randomly selected 10k sets of 141818 (= #Promoter/TSS + #Poised_TSS) regions from the whole set of genomic regions identified by ChromHMM while excluding regions assigned to the superclusters of ‘Promoter/TSS’ or ‘Poised TSS’. A one-sample Wilcoxon signed rank test was applied on the original overlap of the promoter/TSS-like regions with the FANTOM5 TSSs and the overlaps from the random samples. We randomly selected 10k sets of 231848 (#Enhancer) regions from the whole set of genomic regions identified by ChromHMM while excluding regions assigned to the super cluster of ‘Enhancer’. A one-sample Wilcoxon signed rank test was applied on the original overlap of the enhancer-like regions with the FANTOM5 enhancers and the overlaps from the random samples.


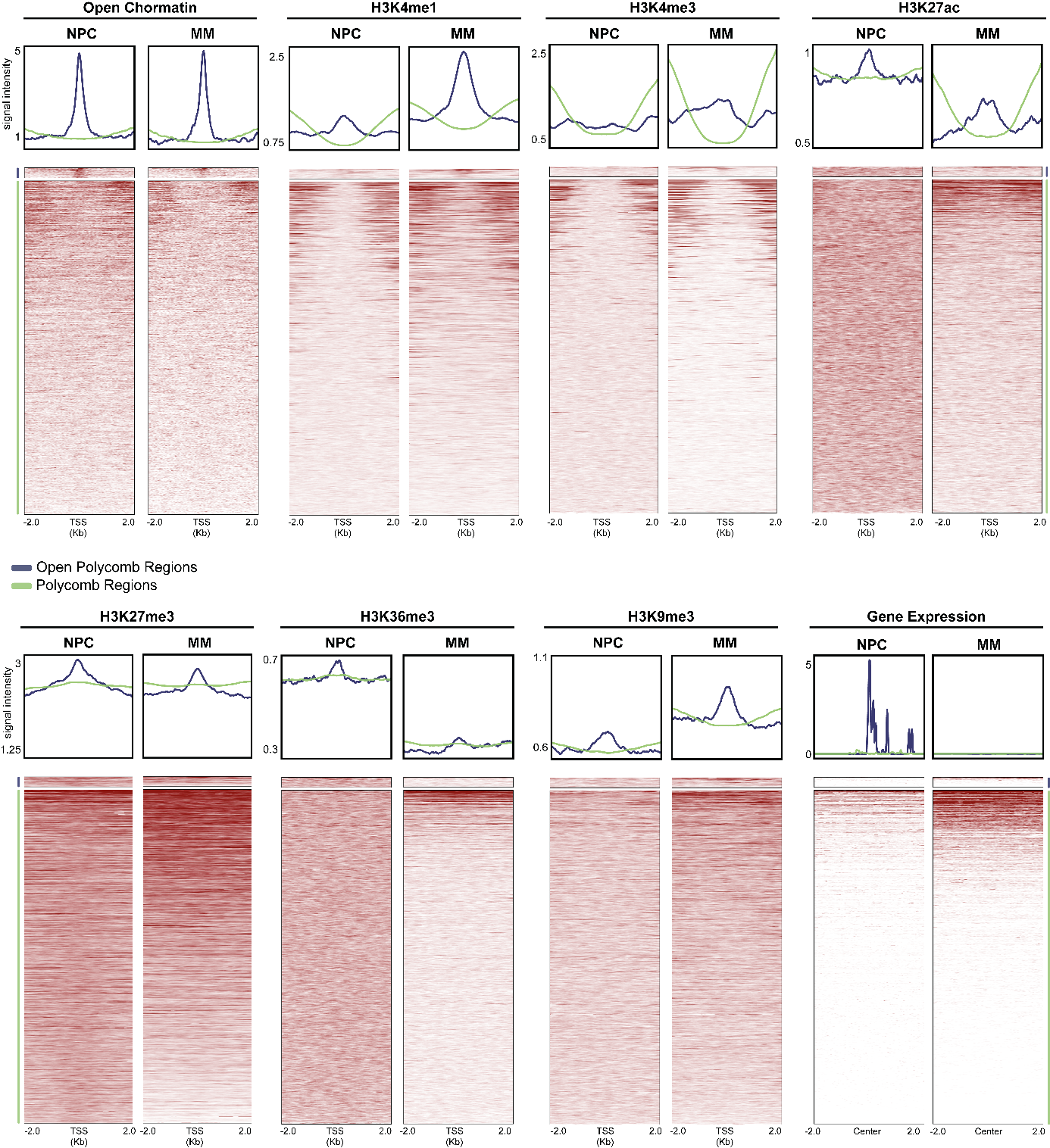


**Supplementary figure 2. MM patients depict redistribution H3K27me3 enrichment in regions defined as Polycomb targets in NPC.** Top, average signal of ATAC-seq, ChIP-seq or RNA-seq reads aligned around the centre of genomic regions belonging to the cluster family defined as polycomb regions. The colour of the lines represents regulatory clusters. Bottom, signal-intensity-ordered heatmaps of each mark in NPC and MM, aligned around the centre of genomic regions.


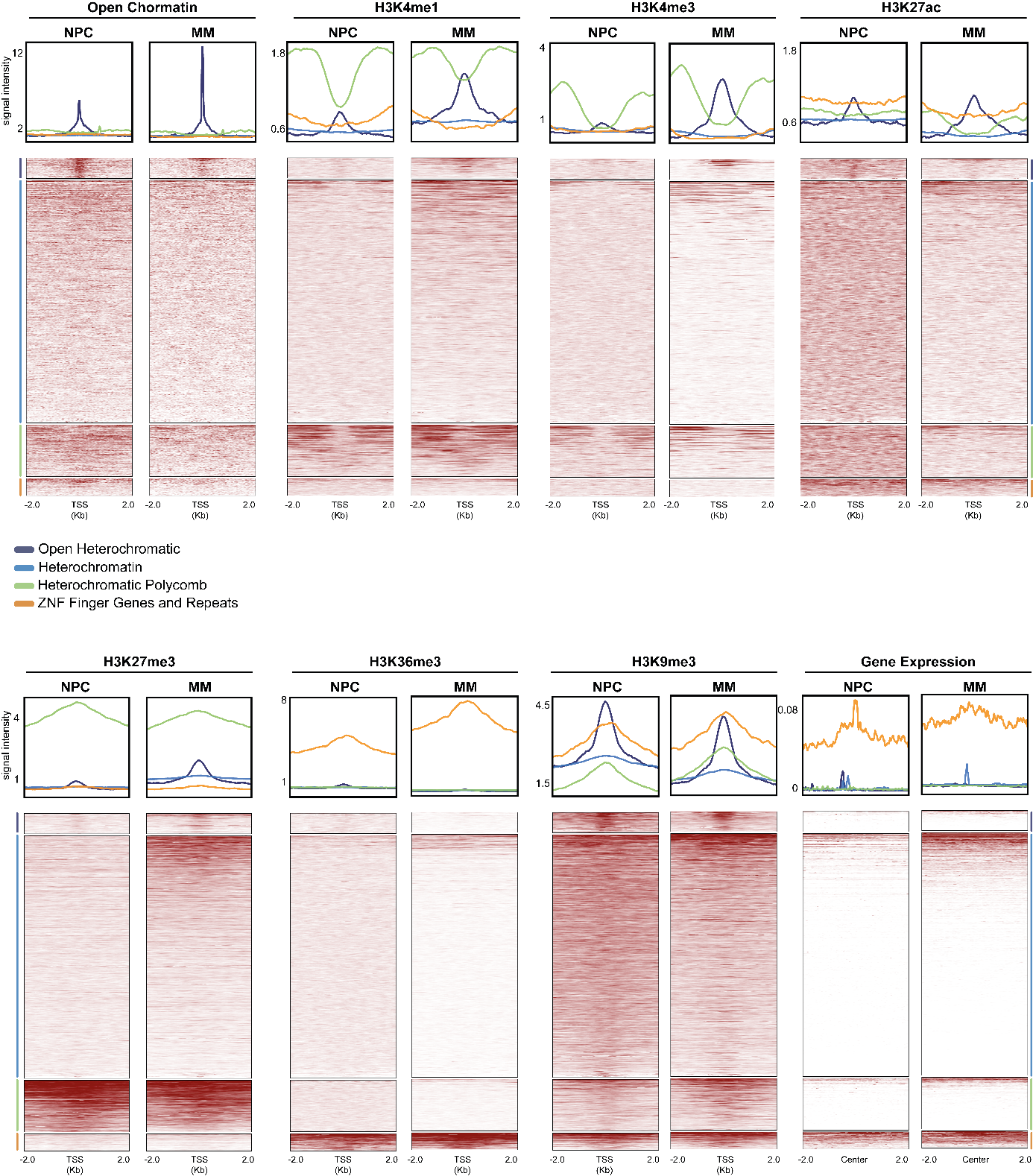


**Supplementary figure 3.** **Heterochromatic regions, initially bearing H3K9me3 in NPC, exchanged this mark for H3K27me3 in MM.** Top, average signal of ATAC-seq, ChIP-seq or RNA-seq reads aligned around the centre of genomic regions belonging to the cluster family of heterochromatin. The colour of the lines represents regulatory clusters. Bottom, signal-intensity-ordered heatmaps of each mark in NPC and MM, aligned around the centre of genomic regions.


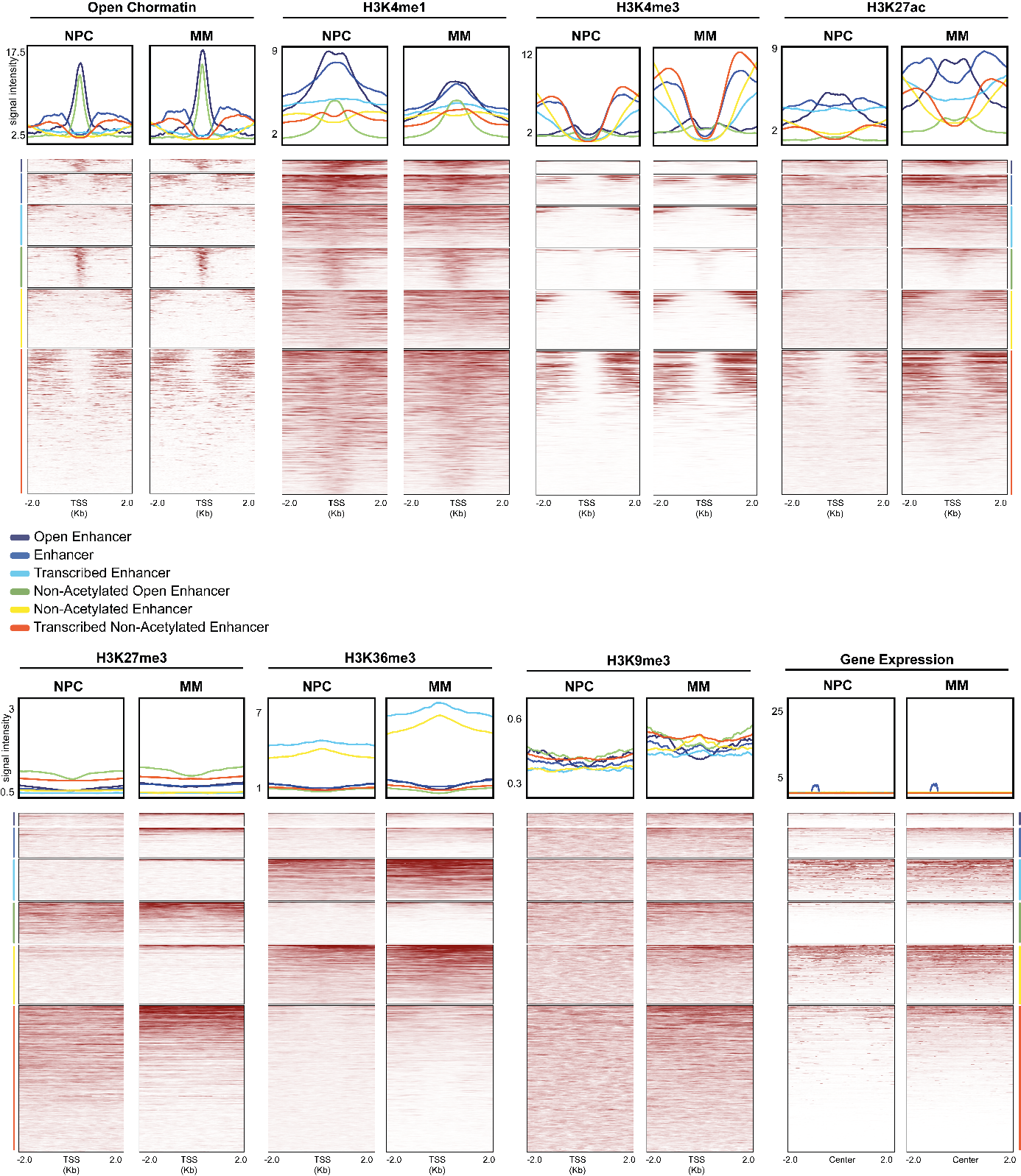


**Supplementary figure 4. Some regions defined as enhancers in NPC gain H3K27me3 and lose their activation marks in MM patients.** Top, average signal of ChIP-seq, ATAC-seq or RNA-seq reads aligned around the centre of genomic regions belonging to the cluster family of enhancers. The colour of the lines represents regulatory clusters as indicated. Bottom, signal-intensity-ordered heatmaps of each mark in NPC and MM, aligned around the centre of genomic regions.


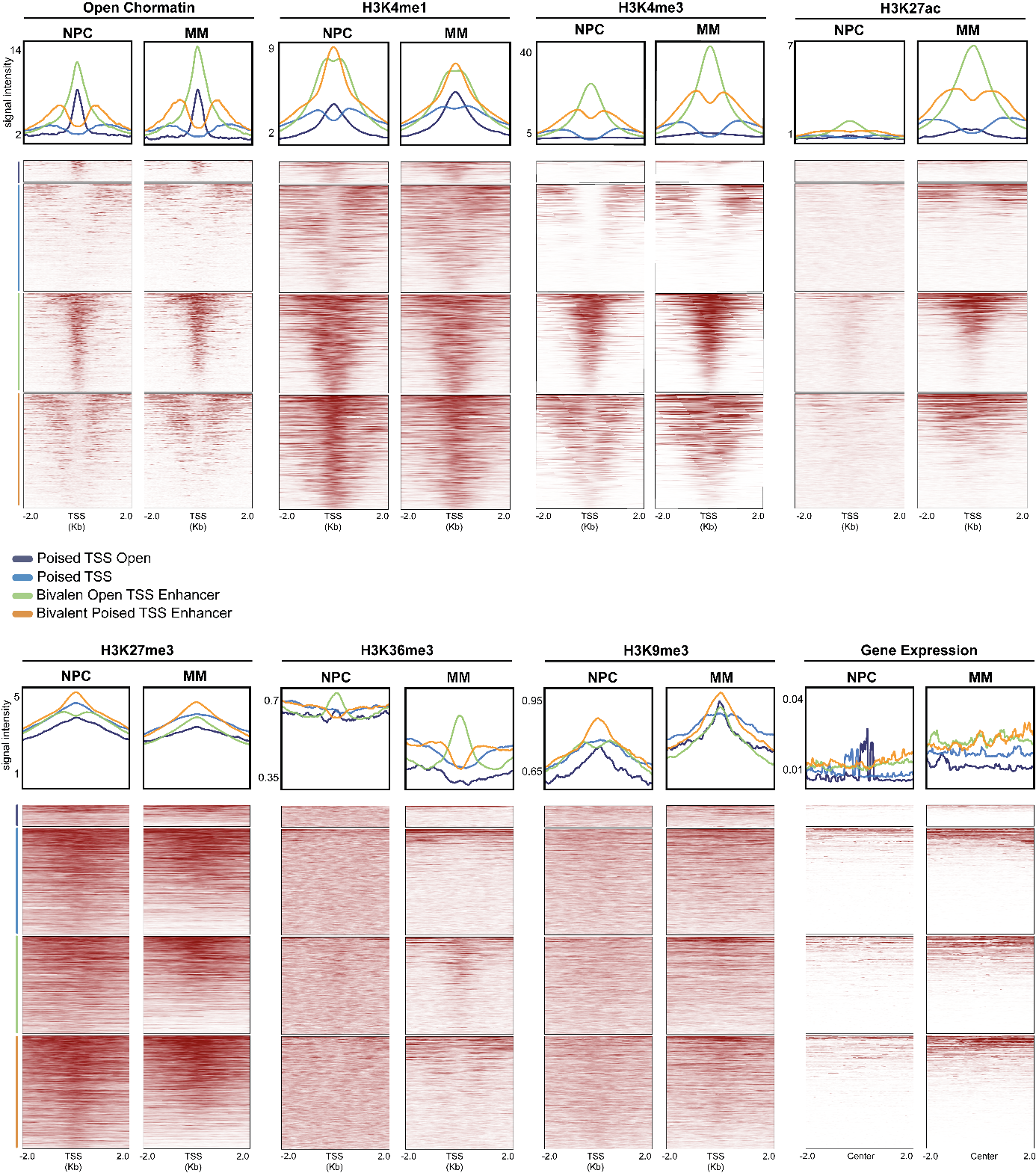


**Supplementary figure 5. Bivalent open TSS enhancers and bivalent poised TSS enhancers gain H3K27ac in MM.** Top, average signal of ATAC-seq, ChIP-seq or RNA-seq reads aligned around the centre of genomic regions belonging to the cluster family of poised TSS/Enhancers. The colour of the lines represents regulatory clusters. Bottom, signal-intensity-ordered heatmaps of each mark in NPC and MM, aligned around the centre of genomic regions.


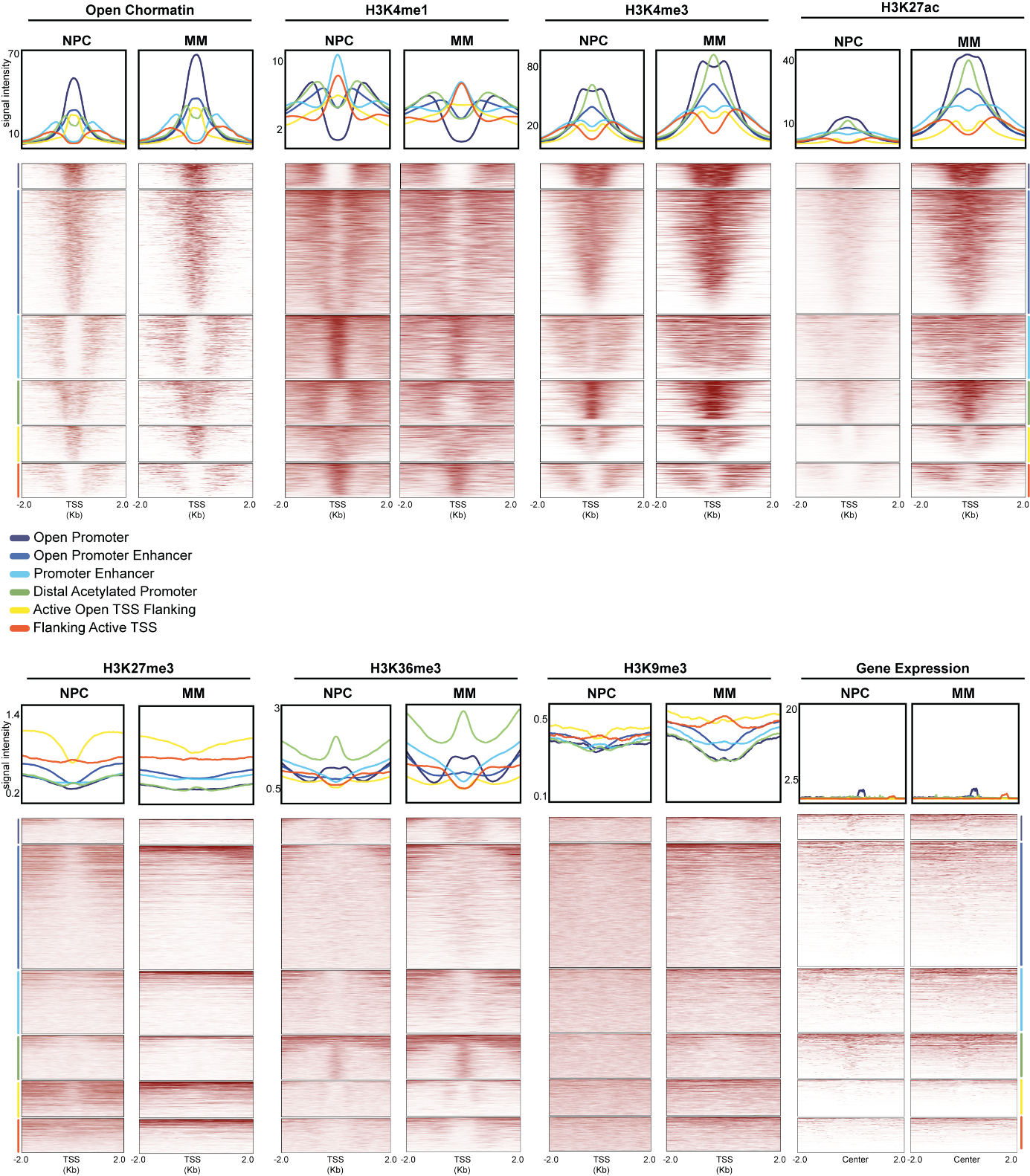


**Supplementary figure 6. Widespread increase of H3K27ac enrichment and targeted redistribution of H3K27me3 in regions defined as active TSS and promoter regions in MM.** Top, average signal of ATAC-seq, ChIP-seq or RNA-seq reads aligned around the centre of genomic regions belonging to the cluster family defined as Promoter/TSS regions. The colour of the lines represents regulatory clusters. Bottom, signal-intensity-ordered heatmaps of each mark in NPC and MM, aligned around the centre of genomic regions.


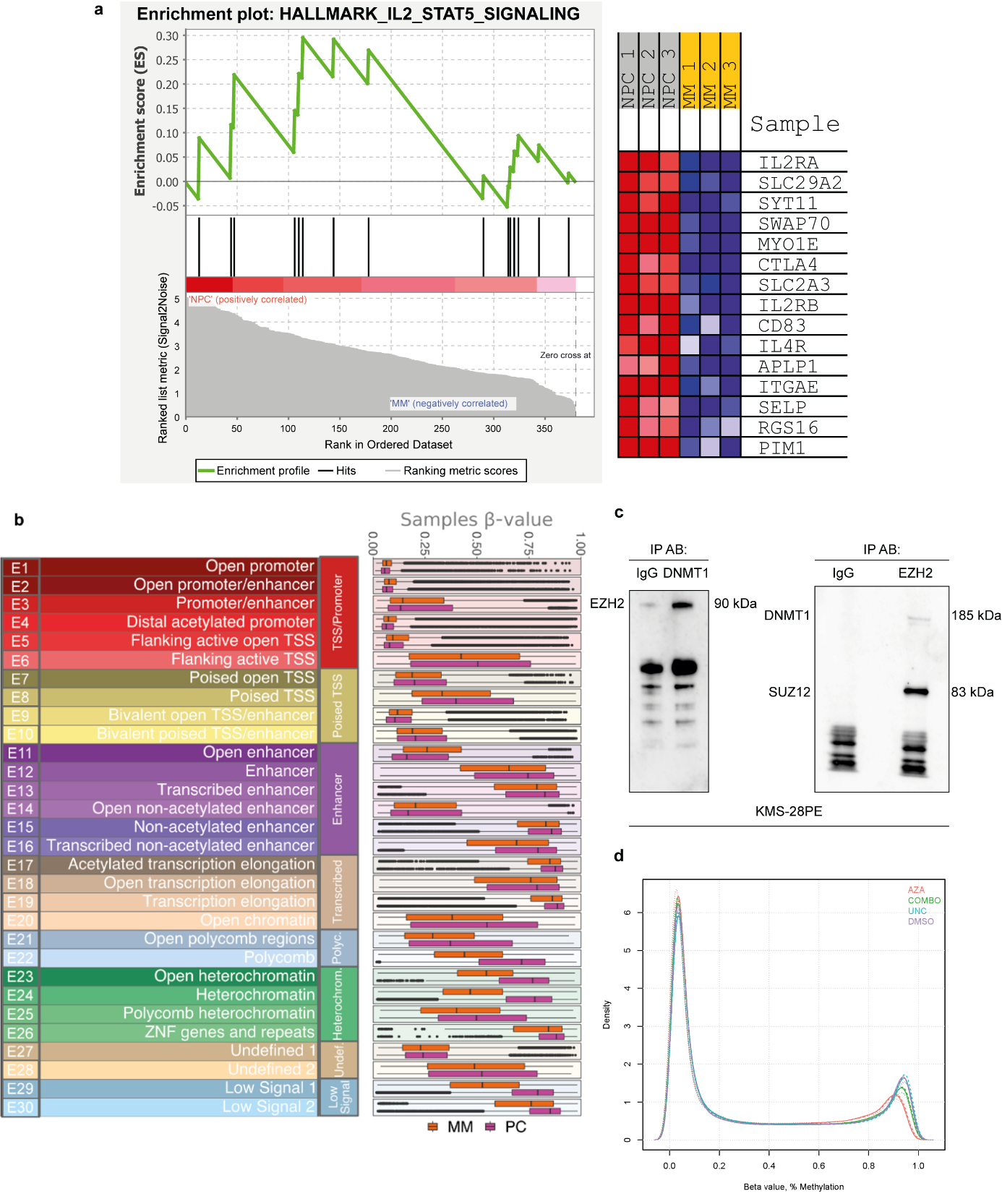
**Supplementary figure 7.** **GSEA of PRC2 target genes in MM patients shows loss of expression in genes associated with IL-2-STAT5 signalling.** **(a)** GSEA of PRC2 target genes in MM. **(b)** Polycomb and Heterochromatin cluster families showed a lower DNA methylation level in MM while certain enhancer clusters are hypermethylated. The figure shows average DNA methylation levels of single clusters. The right panel shows the comparison of the average DNA methylation levels of NPC and MM subjects. **(c)** Co-IP of EZH2-DNMT1 protein-protein interaction. Left panel: DNMT1 IP blotted for EZH2. Right panel: EZH2 IP blotted for DNMT1. SUZ12 was used as a control of interactions. Uncropped representative image can be found in supplementary figure 15a-b. **(d)** DNA methylation beta values in the INA-6 cell line upon treatment with UNC1999 and 5-azacytidine alone or in combination.


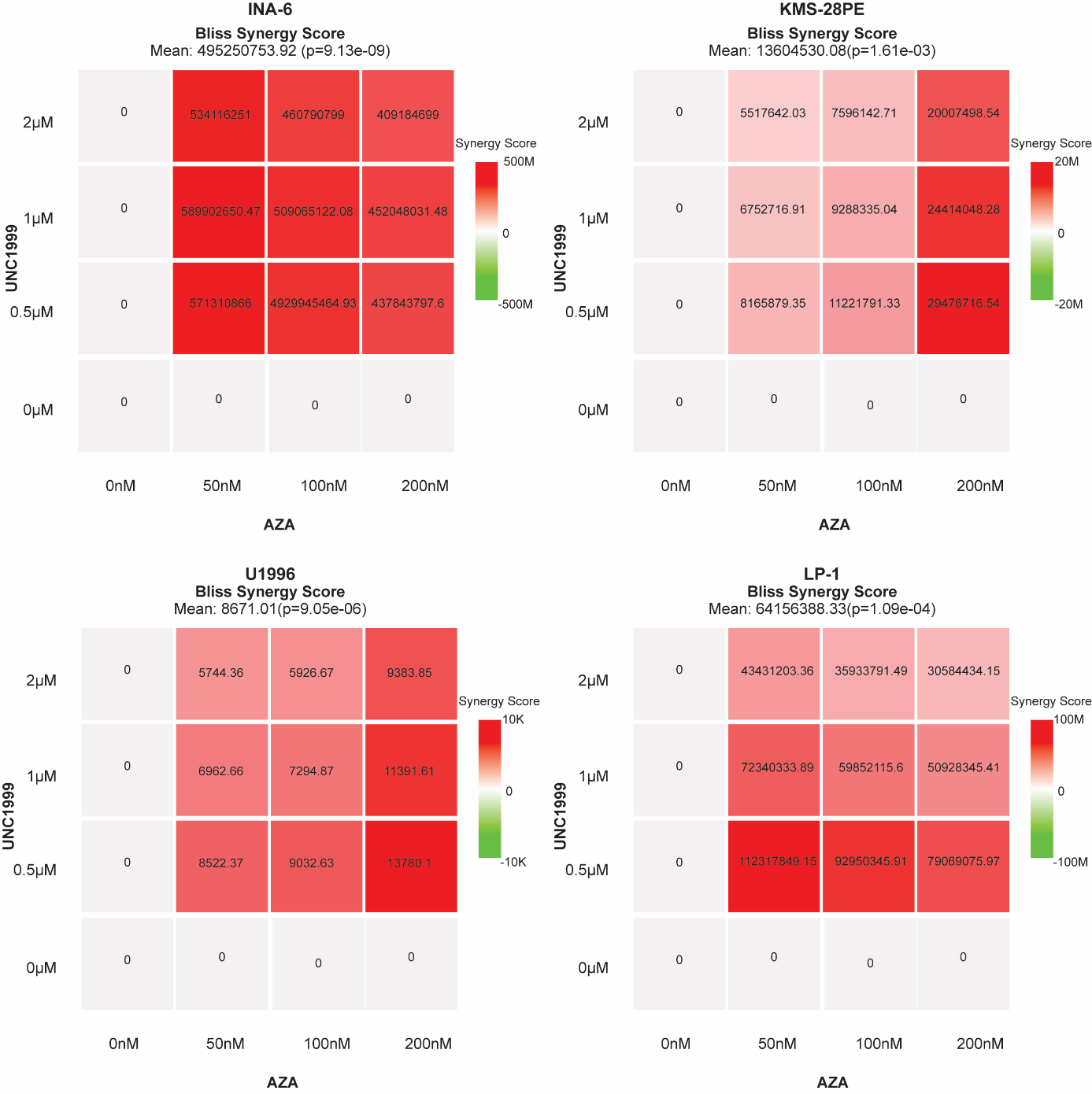


**Supplementary figure 8. AZA and UNC1999 synergy scores in a panel of MM cell lines.** Bliss scores calculated from the effect of the drug combination on viability in INA-6, KMS-28PE, U1996 and LP-1. Bliss score >0 indicates synergy.


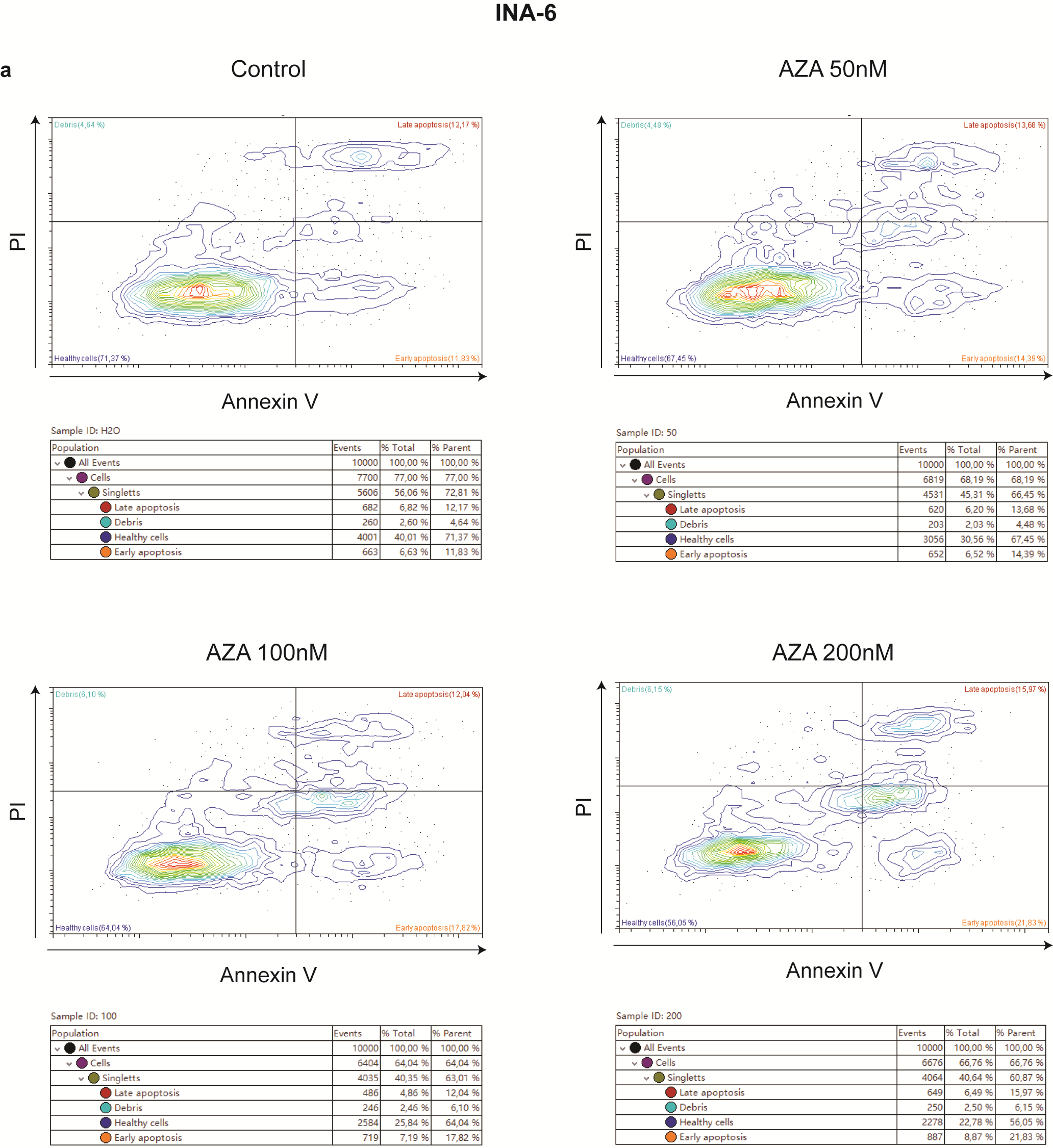


**Supplementary figure 9. Treatment with 5-Azacytidine showed increased apoptosis at a concentration of 200 nM, corresponding to data found in figure 1.** Representative image of sample gating for apoptosis defined by Annexin-V/PI staining in INA-6 MM cells.

**
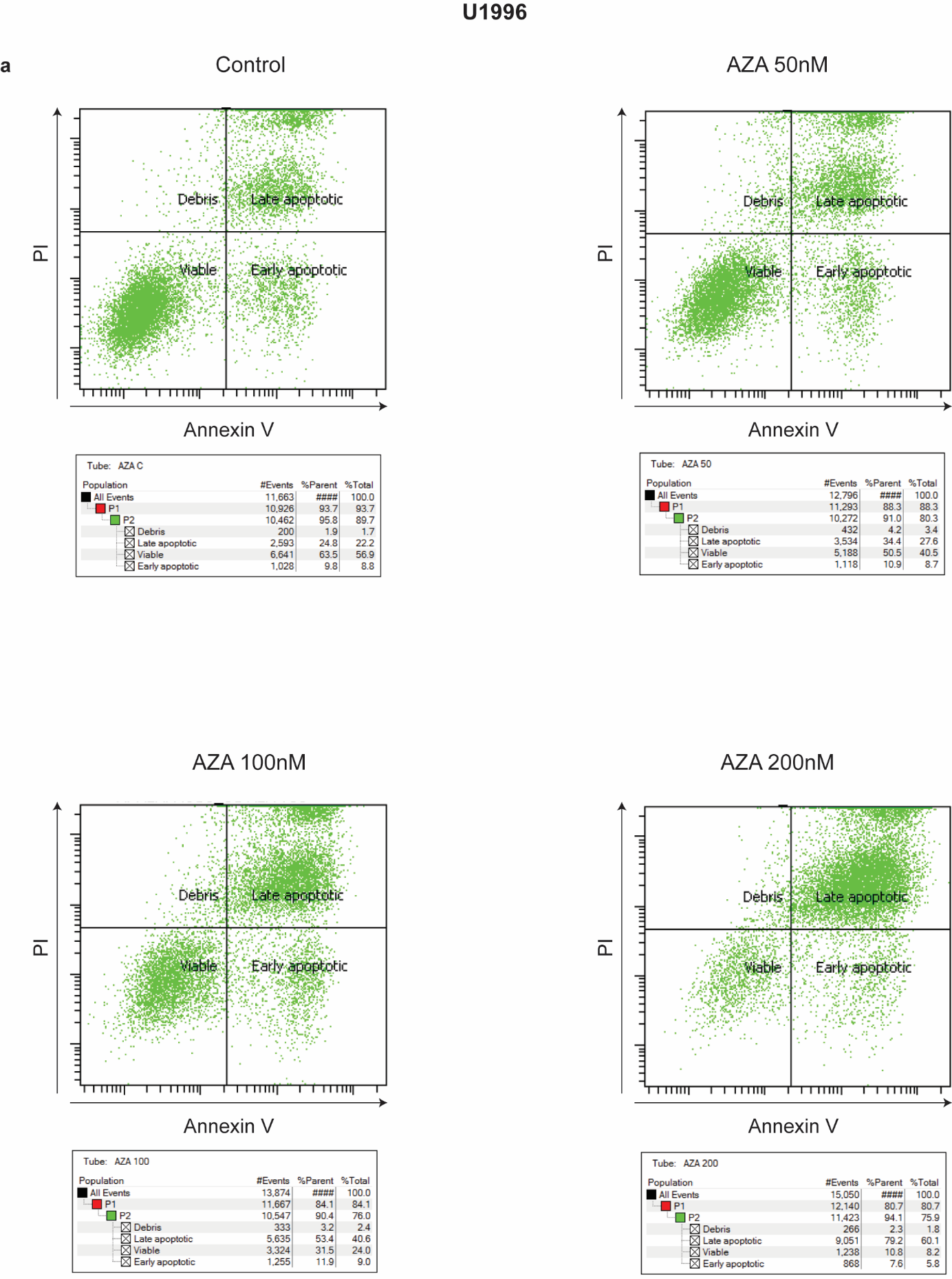
**

**Supplementary figure 10.** **Treatment with 5-Azacytidine showed increased apoptosis at a concentration of 200 nM, corresponding to data found in figure 1.** Representative image of sample gating for apoptosis defined by Annexin-V/PI staining in U1996 MM cells.


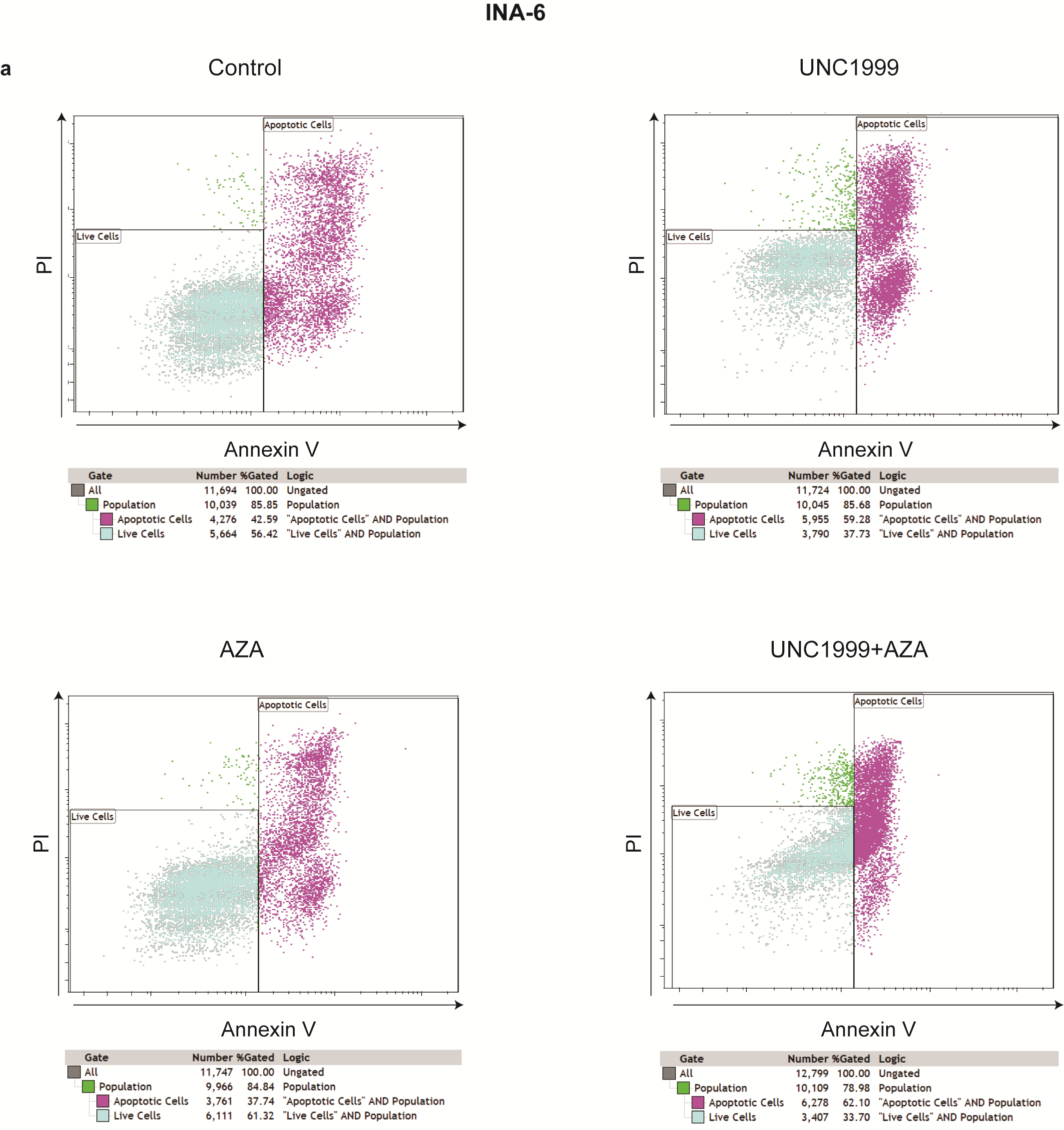


**Supplementary figure 11. Combinatorial treatment with UNC1999 and 5-Azacytidine showed increased apoptosis, corresponding to data found in figure 7.** Representative image of sample gating for apoptosis defined by Annexin-V/PI staining in INA-6 MM cells.

**
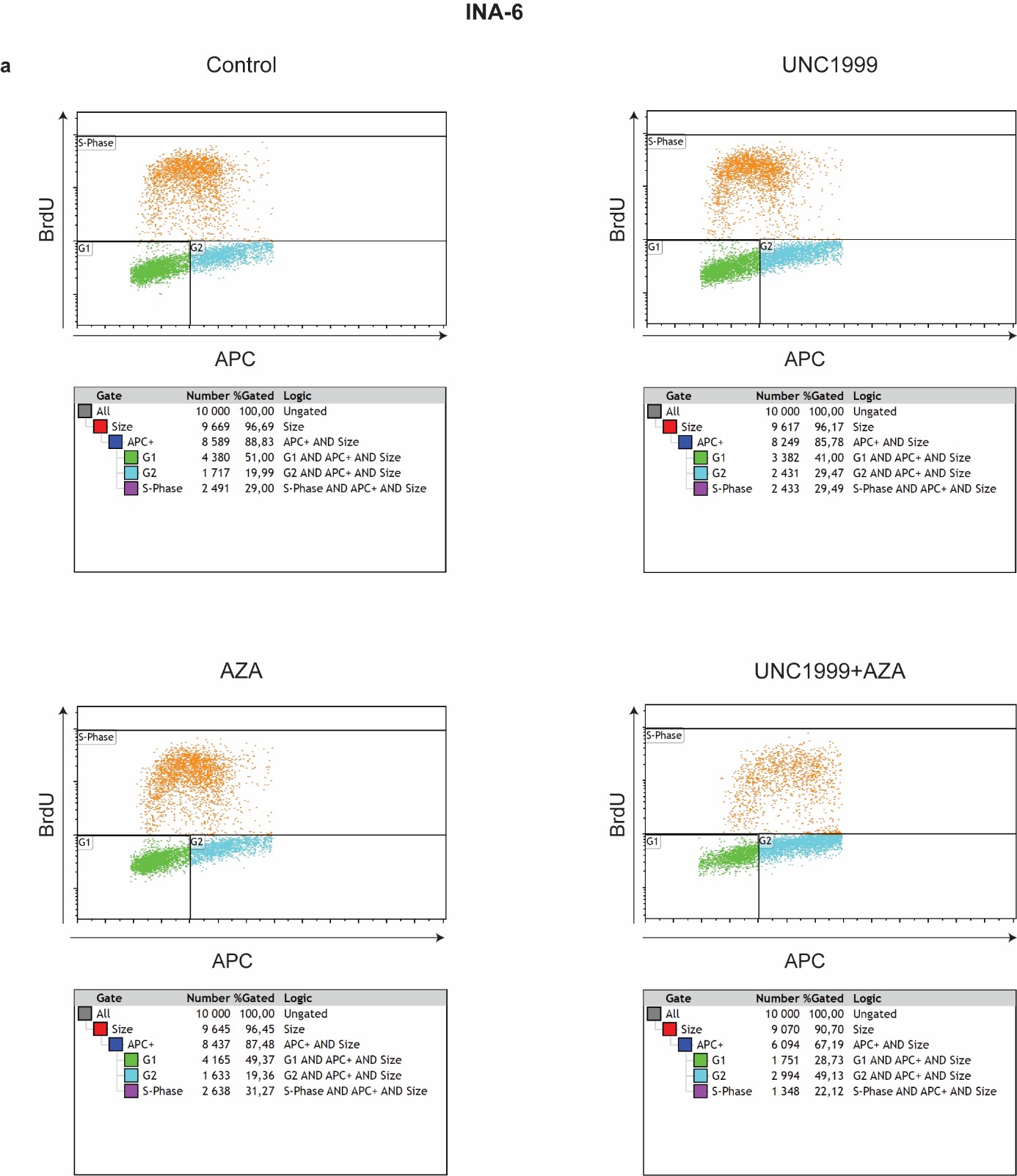
**

**Supplementary figure 12.** **Combinatorial treatment with UNC1999 and 5-Azacytidine showed increased G2/M arrest, corresponding to data found in figure 7.** Representative image of sample gating for cell cycling defined by BrdU/APC staining in INA-6 MM cells.


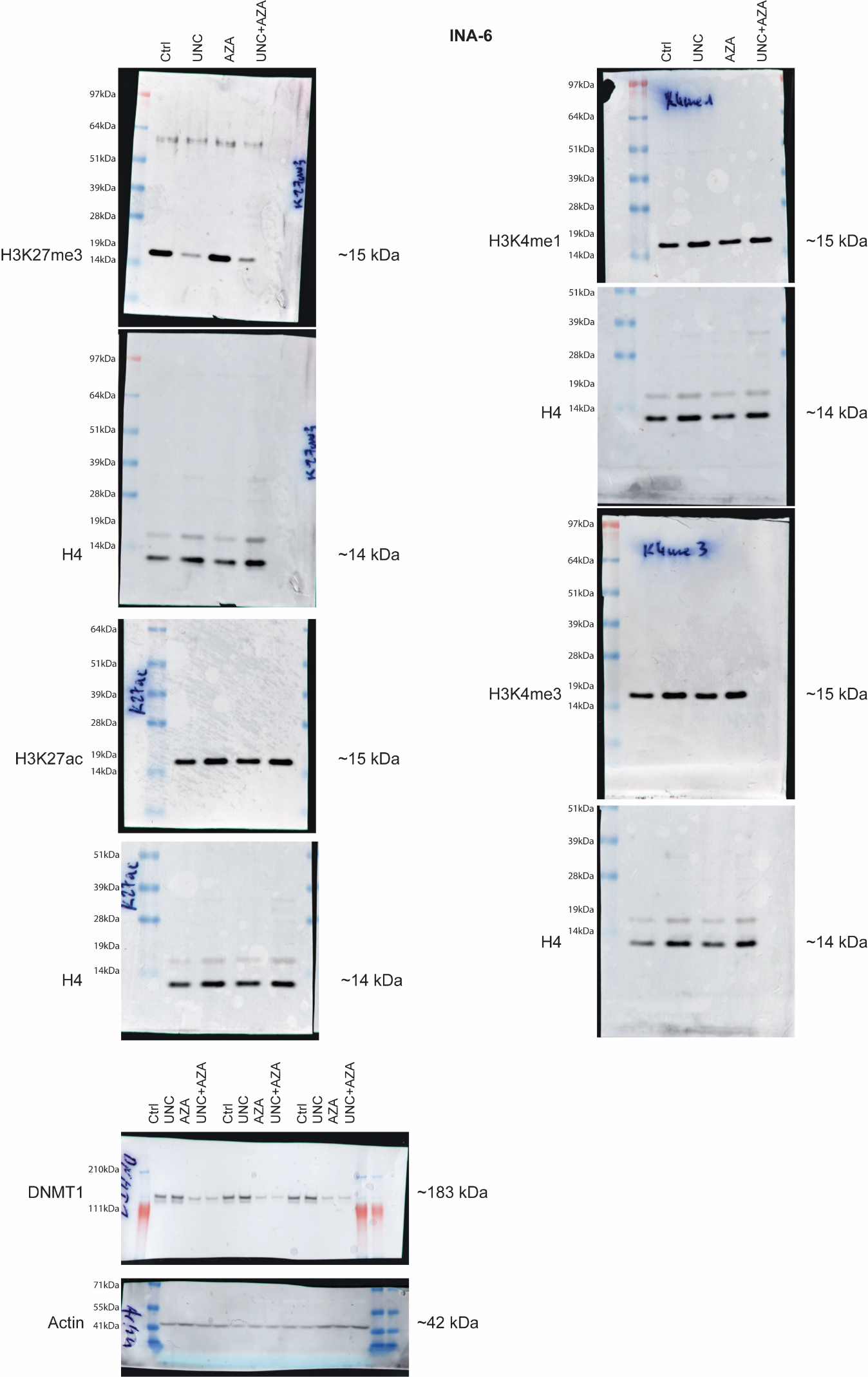


**Supplementary figure 13.** **Representative uncropped western blots of INA-6 cells treated with the combination of UNC1999 and 5-Azacytidine, corresponding to data found in figure 7.** The SeeBlue Plus2 Pre-Stained Standard (Invitrogen; cat. no LC5925) was used as molecular weight marker.


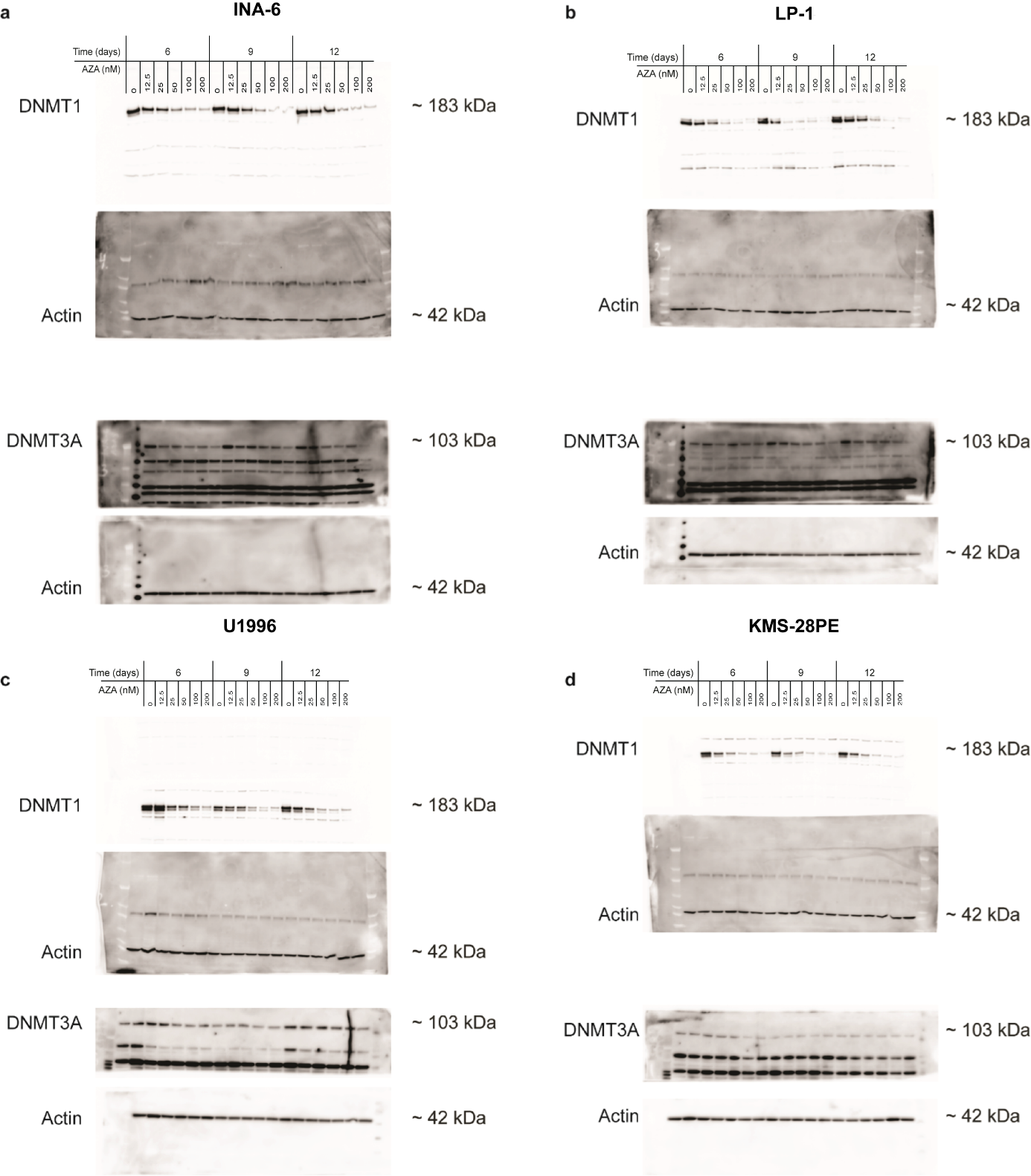


**Supplementary figure 14.** **Representative uncropped western blots of DNMT1 and DNMT3A in INA-6, LP-1, U1996 and KMS-28PE cells treated with 5-Azacytidine, corresponding to data found in Supplementary figure 1.** The SeeBlue Plus2 Pre-Stained Standard (Invitrogen; cat. no LC5925) and the SuperSignal Molecular Weight Protein Ladder (Invitrogen; cat.no 84785) were used as molecular weight markers.


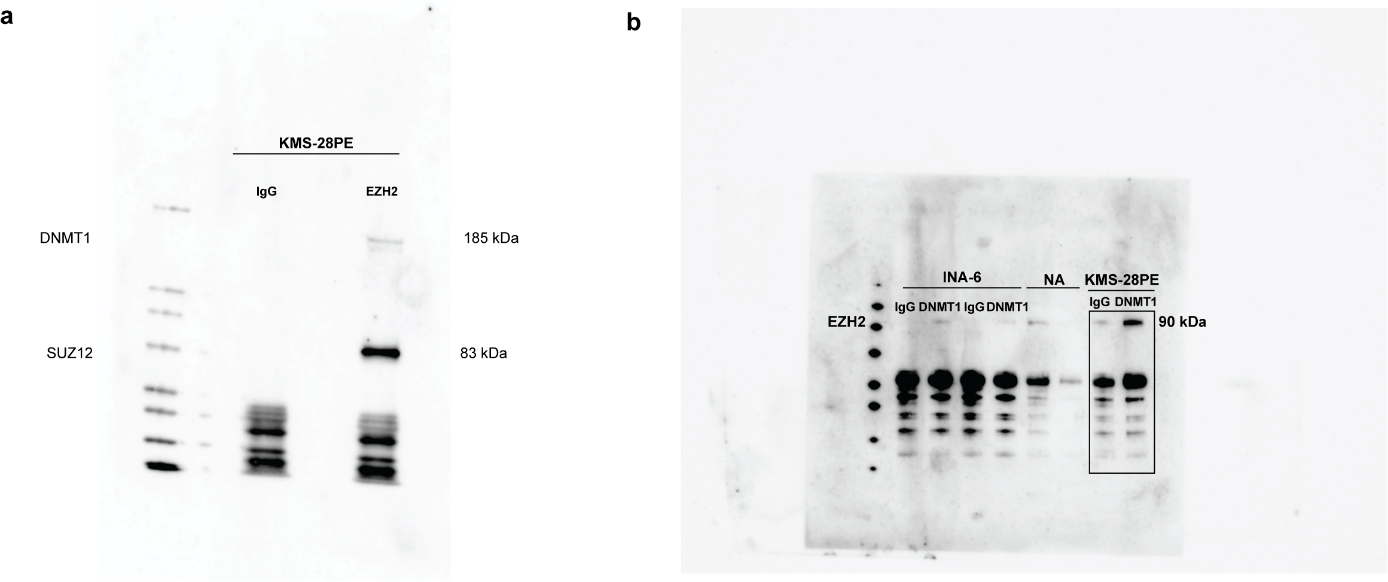


**Supplementary figure 15. Uncropped co-IP blots corresponding to supplementary figure 7c. (a)** Uncropped co-IP blot for EZH2 or IgG IP and blotting for DNMT1 and SUZ12 in KMS-28PE MM cell line. **(b)** Uncropped co-IP blot for DNMT1 or IgG IP and blotting for EZH2 in KMS-28PE MM cell line. Black rectangle depicts replicate included in supplementary figure 7c.
